# Revealing the MRI‐Contrast in Optically Cleared Brains

**DOI:** 10.1002/advs.202400316

**Published:** 2024-04-22

**Authors:** Shimrit Oz, Galit Saar, Shunit Olszakier, Ronit Heinrich, Mykhail O. Kompanets, Shai Berlin

**Affiliations:** ^1^ Department of Neuroscience Faculty of Medicine Technion‐Israel Institute of Technology Haifa 3525433 Israel; ^2^ Biomedical Core Facility Faculty of Medicine Technion‐Israel Institute of Technology Haifa 3525433 Israel; ^3^ L.M. Litvinenko Institute of Physico‐Organic Chemistry and Coal Chemistry National Academy of Sciences of Ukraine Kyiv Ukraine

**Keywords:** brain, lipids, MRI‐contrast, multimodal‐imaging, tissue‐clearing

## Abstract

The current consensus holds that optically‐cleared specimens are unsuitable for Magnetic Resonance Imaging (MRI); exhibiting absence of contrast. Prior studies combined MRI with tissue‐clearing techniques relying on the latter's ability to eliminate lipids, thereby fostering the assumption that lipids constitute the primary source of ex vivo MRI‐contrast. Nevertheless, these findings contradict an extensive body of literature that underscores the contribution of other features to contrast. Furthermore, it remains unknown whether non‐delipidating clearing methods can produce MRI‐compatible specimens or whether MRI‐contrast can be re‐established. These limitations hinder the development of multimodal MRI‐light‐microscopy (LM) imaging approaches. This study assesses the relation between MRI‐contrast, and delipidation in optically‐cleared whole brains following different tissue‐clearing approaches. It is demonstrated that uDISCO and ECi‐brains are MRI‐compatible upon tissue rehydration, despite both methods’ substantial delipidating‐nature. It is also demonstrated that, whereas Sca*l*e‐clearing preserves most lipids, Sca*l*e‐cleared brain lack MRI‐contrast. Furthermore, MRI‐contrast is restored to lipid‐free CLARITY‐brains without introducing lipids. Our results thereby dissociate between the essentiality of lipids to MRI‐contrast. A tight association is found between tissue expansion, hyperhydration and loss of MRI‐contrast. These findings then enabled us to develop a multimodal MRI‐LM‐imaging approach, opening new avenues to bridge between the micro‐ and mesoscale for biomedical research and clinical applications.

## Graphical abstract

We challenged the dogma regarding lipids as the primary source for ex vivo brain MRI contrast. By assessing clearing methods with MRI and lipidomic analysis, we distinguished lipid content from contrast. We revealed a link between tissue expansion, hyperhydration, and reduced contrast. Restoring MRI contrast to cleared brains enabled multimodal imaging of entire brain with MRI and light microscopy.

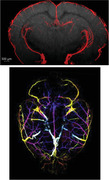

## 1 Introduction

Magnetic Resonance Imaging (MRI) is a powerful imaging technique that is suited to explore the intricate structures of the brain at the mesoscale, non‐invasively, particularly owing to its soft tissue‐compatibility. Remarkably, MRI generates brain images with sufficient contrast obviating the need for exogenous contrast‐agents.^[^ ^1^ ^]^

MRI is built upon the principles of nuclear magnetic resonance, a phenomenon in which the nuclei of certain atoms, typically water hydrogens, interact with magnetic fields and radio waves to produce an MRI‐signal. MRI‐contrast is obtained by regional variations in these interactions that may result from inhomogeneous distribution of hydrogen atoms in the tissue and/or variations in hydrogen atoms’ interactions with adjacent molecules in the tissue. The latter leads to differential rates at which hydrogen nuclei return to their equilibrium state (i.e., T1: spin‐lattice and T2: spin‐spin relaxation times), translating into detectable MRI‐contrast in images.^[^ ^1^ ^]^

The role and contribution of various molecules in the brain to MRI‐contrast have been extensively explored, although the exact biophysical basis for the observed contrast remains debated.^[^ ^2^, ^3^ ^]^ For instance, brain features such as fiber orientation,^[^ ^4^ ^]^ blood,^[^ ^5^ ^]^ inorganic ions,^[^ ^6^ ^]^ lipids^[^ ^7^ ^]^ and other pervasive macromolecules and proteins^[^ ^8^ ^]^ are known to influence MRI‐contrast. Nonetheless, ongoing discussions surround the debate over the relative impact and essentiality of these elements (see, e.g., refs. [3, 7, 9, 10, 11, 12, 13, 14, 15, 16] vs [17, 18, 19, 20, 21, 22]). The debate recently heightened due to a report proposing that lipids play an exclusive role in MRI‐contrast, largely dismissing the relevance of all other elements.^[^ ^2^ ^]^ The reason behind these discrepancies and uncertainties largely stems from challenges in dissection of the tissue into its basic elements, particularly lipids. However, the advent of tissue‐clearing techniques has provided means to overcome some of these challenges, enabling a more comprehensive exploration of the role and contribution of lipids to MRI‐contrast.

Tissue‐clearing approaches render biological samples optically transparent,^[^ ^23^ ^]^ thereby enabling to capture detailed 3D structures via advanced light microscopy (LM) approaches (e.g., light‐sheet microscopy; LSM^[^ ^24^ ^]^). Importantly, multiple clearing approaches achieve transparency by removal of lipids (i.e., delipidation) from the tissue. Thus, these techniques appear to be ideal for addressing, at least, the contributions of lipids toward MRI‐contrast under ex vivo settings (here, denoted ex vivo MRI).^[^ ^2^, ^25^, ^26^, ^27^ ^]^ Indeed, a recent report has leveraged tissue clearing and demonstrated that optically cleared brains do not show any ex vivo MRI‐contrast and, thereby, proposed that lipids are exclusively responsible for MRI‐contrast.^[^ ^2^ ^]^

Whereas the hypothesis attributing MRI‐contrast to lipids has been gaining some acceptance (see, e.g., refs. [25, 26, 28]), it remains controversial for obvious reasons. 1) It deviates from a substantial body of literature that highlights other features in the brain as significant sources for MRI‐contrast. 2) Only a handful of clearing methods have been studied in conjunction to ex vivo MRI. Consequently, the findings are limited to a specific method, experimental settings, reagents, and conditions that may also influence MRI‐contrast, independent of lipid content. 3) To the best of our knowledge, no lipid‐retaining clearing method (i.e., a clearing method that does not depend on extrusion of lipids to achieve transparency—non‐delipidating) has been combined with ex vivo MRI to firmly establish the role of lipids. 4) The potential of restoring MRI‐contrast to cleared samples has not been previously explored.

Here, we addressed these gaps by studying ex vivo MRI‐contrast in cleared whole brains produced by four different clearing methods, including delipidating and non‐delipidating methods. Collectively, our results show no association between lipid‐content and MRI‐contrast. We also demonstrate that MRI‐contrast can be restored to brains that were specifically cleared by the CLARITY method (CLARITY is the method previously employed for establishing the debated association between lipids and MRI‐contrast). These strongly argue against a major role for lipids in ex vivo MRI‐contrast. Instead, we show that MRI‐contrast is masked by excessive hyperhydration and tissue expansion of the samples. We then harness these observations to uniquely demonstrate the feasibility of performing multimodal LM‐MRI of cleared intact brains.

## 2 Results

### 2.1 Solvent‐Based Clearing Methods Yield Cleared Brains with No MRI‐Contrast

**Figure 1:**
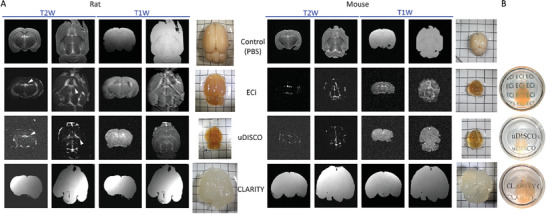
Cleared brains by three clearing approaches lack MRI‐contrast. A) Coronal and horizontal T2W and T1W MRI of rat (left) and mouse (right) whole brains. Brains were PFA‐fixed and maintained in PBS (top row, Control), or cleared via ECi, uDISCO or CLARITY approaches (lower panels). All samples were imaged in Fomblin Y. White arrowheads point to loci exhibiting visible MRI‐signal, such as in brain ventricles. Images of brain samples (before incubation in RI‐matching solution), are shown on the right (placed on a 0.5‐cm grid paper). B) Images of transparent ECi‐, uDISCO‐ and CLARITY‐mouse brains immersed in their corresponding RI‐matching solutions.

We cleared whole intact rodent brains (rats and mice) by two solvent‐based tissue‐clearing methods, ECi^[^ ^29^ ^]^ and uDISCO^[^ ^30^ ^]^, and by the hydrogel embedding tissue‐clearing protocol denoted CLARITY.^[^ ^31^ ^]^ ECi and uDISCO‐cleared brains (ECi‐ and uDISCO‐brains, in brief) appear amber‐colored, translucent, and of reduced size; with ECi‐ and uDISCO‐brains undergoing 40% and 60% shrinkage (**Figures** **1**A; right, **2**A).^[^ ^30^ ^]^ Inversely, CLARITY‐brains expanded in size quite significantly (250%), appearing yellowish, gelatinous and hyperhydrated (Figures 1A; bottom, 2A)^[^ ^32^ ^]^ When immersed in their corresponding refractive‐index (RI)‐matching solutions, all specimens attained a high degree of transparency (**Figure** **1**B). Of note, all features observed were previously described, suggesting that we have attained equal clearing performances.^[^ ^30^, ^32^ ^]^ We proceeded to image cleared brains by T1‐ and T2‐weighted (T1W and T2W) MRI sequences. Control PFA‐fixed brains (maintained in PBS) showed characteristic MRI‐contrast (Figure 1A), whereas CLARITY‐brains showed very bright and homogenous MRI‐signals across the entire tissue with complete absence of MRI‐contrast, as initially reported (Figure 1A)^[^ ^2^, ^25^ ^]^ Imaging of uDISCO‐ and ECi‐brains by MRI yielded very blurred images, owing to minute signals. Importantly, these images were devoid of obvious MRI‐contrast as seen in control brains (especially in mice brains), except for discrete regions in the tissue, such as ventricles (Figure 1A; arrowheads; Figure S1A, Supporting Information). Even a large brain structure such as the corpus callosum (easily observed in control samples) does not appear in uDISCO‐ or ECi‐cleared brain samples. Thus, CLARITY‐, uDISCO‐ and ECi‐brains produce no detectable MRI‐contrast. However, unlike CLARITY, uDISCO‐ and ECi‐brains also lack an MRI‐signal.

**Figure 2:**
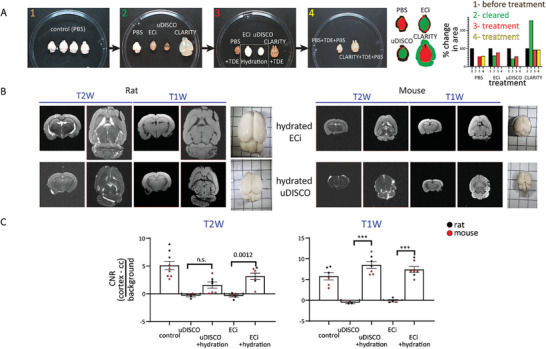
Retrieval of MRI‐contrast in ECi‐ and uDISCO‐brains. A) Four PFA‐fixed mouse brains were imaged at different time points, to demonstrate the longitudinal changes in appearance and size following clearing and hydration procedures. Brains are placed in a 10‐cm dish (captures 1–4, above). The areas of the brains, captured from a top view after each treatment, were illustrated when these are superimposed. The change in the calculated area per treatment, of each brain sample, was normalized to control before treatment, shown as % change. B) Coronal and horizontal T2W and T1W MRI images of rat (left) and mouse (right) whole brains following hydration by PBS. Right columns, images of the hydrated brains, placed on a 0.5‐cm grid paper. C). Summary of contrast‐to‐noise ratio (CNR). CNR values from coronal T1W and T2W MRI of mouse (red) and rat (black) were calculated from the difference between signal intensities obtained in the cortex (gray matter) and the corpus callosum (cc, white matter), divided by the background signal. Each point represents one animal's whole brain. Data are presented as mean ± SEM. One‐way ANOVA, following Sidak *post‐hoc* test, was used to determine statistical significance between treatments. P‐values are indicated; ^***^, *p* < 0.001, n.s., non‐significant.

### 2.2 Hydration of Solvent‐Cleared Brains Restores MRI‐Contrast

The lack of MRI‐signal in uDISCO‐ and ECi‐brains suggested a complete dehydration of the tissue, as would be expected by a solvent‐based clearing procedure. We assumed that re‐hydration of the tissues may restore the signal. We thereby immersed uDISCO‐ and ECi‐brains in PBS for three days. Hydration of the cleared brains changed their appearances, rendering them opaque white, with very subtle changes in volume (**Figure** **2**A). Importantly, hydration of uDISCO‐ and ECi‐brains restored the MRI‐signal and, surprisingly, MRI‐contrast (Figure 2B; Figure S1B, Supporting Information). The visible MRI‐contrast presented variable contrast‐to‐noise ratio (CNR) values across different brain regions, which allowed for comprehensible analysis of the brains’ anatomy (Figure 2C; Figure S2, Supporting Information). Of note, MRI‐contrast was particularly strong in T1W images, with CNR values on par with those of controls, whereas T2W images showed moderate, albeit significant, increases in CNR (Figure 2C; Figure S2C, Supporting Information). We also examined longer hydration durations, although maximal CNR was obtained following three days, regardless of whether the cleared brains were imaged in PBS or Fomblin Y, namely with high or low background signals, respectively (Figure S3, Supporting Information). These observations are also demonstrated in the T1‐, T2‐ and T2*W maps of hydrated uDISCO‐ and ECi‐brains, with pronounced MRI‐contrast and reduced relaxation times compared to controls (Figure S4 and Table S1, Supporting Information).

We next speculated whether gadolinium‐based contrast‐agents (GBCA) could further enhance the restored contrast. Briefly, GBCA amplifies the extant MRI‐contrast, provided by the differential distribution and concentration of water and “endogenous contrast‐agents” (e.g., lipids, iron, proteins).^[^ ^3^ ^]^ We locally injected GBCA to one hemisphere of hydrated uDISCO‐ and ECi‐brains, and the saline carrier to the second hemisphere as control. T1W images were acquired several minutes after GBCA injection, and these showed an enhanced MRI‐signal and contrast exclusively at the site of injection. Conversely, GBCA injections to CLARITY‐brains yielded very small increases in MRI‐signal, but with no improvement in MRI‐contrast (Figure S5, Supporting Information). We also noted that the enhanced signal following GBCA injection was much more diffusive in CLARITY brains, compared to uDISCO‐ and ECi‐brains. This is consistent with previous reports showing that the diffusion and relaxation rates of water molecules within the CLARITY‐brain slices resemble those of free water,^[^ ^2^ ^]^ and with our own observations that CLARITY‐brains are highly expanded (Figure 1) and porous.^[^ ^33^ ^]^ The effect of GBCA on uDISCO‐ and ECi‐brains suggests the presence of “endogenous contrast‐agents” in the tissue that provide the basis for the observed MRI‐contrast. However, the lack of effect of GBCA on MRI‐contrast and MRI‐signal in CLARITY‐brains suggests that the expanded‐ and hyperhydrated‐state of the cleared brain contribute to the excessive and homogenous MRI‐signal across the tissue, thereby obscuring underlying MRI‐contrast.

### 2.3 Recovery of MRI‐Contrast in CLARITY‐Cleared Brains

**Figure 3:**
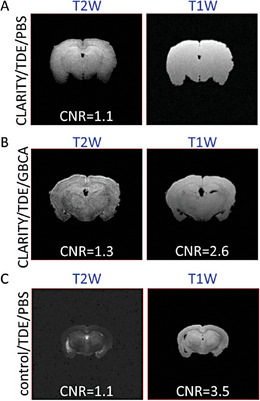
Recovery of MRI‐contrast in CLARITY‐cleared brains. A) T2W and T1W MRI and CNR values for CLARITY‐cleared mice following prolonged TDE incubation and immersion in PBS (A) or when incubated with 5 mm GBCA (B), compared to control samples (C). CNR values are calculated as in Figure 2C.

The recovery of MRI‐contrast in uDISCO‐ and ECi‐brains motivated us to examine whether MRI‐contrast can be similarly restored to CLARITY‐brains. However, unlike uDISCO‐ and ECi‐brains that displayed no MRI‐signal, CLARITY‐brains exhibits excessive MRI‐signal likely due to hyperhydration (Figures 1 and 2A). We thereby immersed CLARITY‐brains in their RI‐matching solution 2,2′‐thiodiethanol (TDE), with the intention to extract water from the samples (Figure 1B, bottom). Overnight immersion of CLARITY‐brains in TDE resulted in a slight decrease in the overall size and MRI‐signal of the sample, however, it revealed no MRI‐contrast (Figures S1B and S6A, Supporting Information, CLARITY‐partial TDE). Longer incubations in TDE caused the complete extrusion of water from the sample and complete loss of MRI‐signal in CLARITY‐ and in control‐brains (Figure S6B,C, Supporting Information), akin to ECi‐ and uDISCO‐brains (Figure 1). We then modified our protocol to immersion of the samples for seven days in TDE for maximal shrinkage, followed by an equal duration in PBS (for hydration). This procedure significantly shrunk controls and CLARITY‐brains, with CLARITY‐brains undergoing the strongest reduction in volume, nearly to their initial size (Figure 2A). TDE‐PBS‐treatment gave rise to detectable MRI‐signals and—importantly—MRI‐contrast in CLARITY‐brains, particularly under T2W sequences (**Figure** **3**A compared to CLARITY‐brain Figure 1A; bottom), however MRI‐contrast in T1W images could be significantly enhanced by the addition of GBCA (Figure 3B). However, the reason for only partial recovery in MRI‐contrast of CLARITY‐brains, compared to control brains that went through identical treatment, is unclear (Figure 3C; Table S1, Supporting Information).

### 2.4 Assessing Iron‐Content in Cleared Brains

The results above suggested that CLARITY‐brains maintain some of the “endogenous contrast‐agents” that can provide MRI‐contrast, particularly under T2W sequences (Figure 3A). One of the most accepted T2W‐relevant “endogenous contrast‐agents” in the brain is iron,^[^ ^18^ ^]^ however whether iron is removed by various clearing processes is largely unknown (see, e.g., refs. [2, 17]). We therefore employed Inductively Coupled Plasma‐Optical Emission Spectrometry (ICP‐OES) to explore remaining iron concentrations only to find that they were not significantly altered after clearing by CLARITY, ECi and uDISCO compared to control samples (Figure S7, Supporting Information). These results are in agreement with the T2* maps that show clear MRI‐contrast in hydrated ECi‐ and uDISCO‐brains (Figure S4 and Table S1, Supporting Information). This suggests that iron may contribute to the MRI‐contrast that we have recovered in CLARITY‐brains, and to the contrast observed in uDISCO‐ and ECi‐brains. However, these concentrations are not sufficient to provide MRI‐contrast in CLARITY‐brains before TDE‐PBS treatment (i.e., before shrinkage).

### 2.5 Qualitative Assessment of Lipids in Cleared Brains

The discrepancies between our observation of extant MRI‐contrast in CLARITY‐cleared brains and previous reports showing absence of MRI‐contrast in cleared‐samples^[^ ^2^, ^25^, ^26^, ^27^ ^]^ and the results with iron (above), raised the possibility of variations in the extent of delipidation of the CLARITY samples. It also raised the possibility that uDISCO‐ and ECi‐brains are perhaps less delipidated than CLARITY‐brains, which would then contribute to the observed MRI‐contrast in these samples. Notably, whereas lipid content was assayed in brain tissue following clearing by CLARITY,^[^ ^2^ ^]^ we found no report to have quantitatively and systematically examined the extent of delipidation by other clearing methods.

We have initially employed a variety of established methods to explore delipidation: 1) electrophoretic tissue‐clearing (ETC) (i.e., the active delipidation step in CLARITY^[^ ^31^, ^34^, ^35^ ^]^), 2) staining with lipidic‐dyes (Oil Red O, ORO and 1,1′‐dioctadecyl‐3,3,3′,3′‐tetramethylindocarbocyanine perchlorate, DiI), and 3) assayed lipid extraction using the DiI‐washout method. However, all trials yielded conflicting results and—importantly—multiple artifacts arising from the use of lipophilic dyes with solvent‐based clearing methods (see our *Data in Brief* report^[^ ^33^ ^]^). We then assessed lipid content by Raman spectroscopy (RS), in particular because this method has been previously employed to explore the lipid‐content and lipid‐types in a cleared specimen.^[^ ^36^, ^37^ ^]^ Briefly, RS can be used to measure the relative composition and abundance of lipids by their characteristic chemical bonds without the need for chemical labeling.^[^ ^38^ ^]^ Though lipids signatures span across the entire RS spectrum, we focused on the ratio between peaks appearing at 2850 cm^−1^ (representing CH_2_ bonds of long aliphatic lipid chains) and 2940 cm^−1^ (attributed to CH_3_ bonds as typically found in proteins) to assess protein/lipid ratio, as previously shown.

Spectra obtained from control brain slices showed the prototypical signature of biomolecules in the brain, with the expected lipids and proteins bands at 2850 and 2940 cm^−1^, respectively (Figure S8A, Supporting Information). We noted that the 2850 and 2940 cm^−1^ peaks ratio was variable between different regions of the slice, however, both peaks remained highly visible (Figure S8A, Supporting Information, inset). We examined CLARITY‐brain slices at two different time points: after partial (3 h ETC) or prolonged clearing (9 h ETC) and observed a progressive reduction in the 2940/2850 cm^−1^ ratio; indicative of the relative reduction in proteins compared to lipids (Figure S8B, Supporting Information). Analysis of ECi‐ and uDISCO‐brain slices showed substantial contribution of the solvents to the spectra, and these overlapped with the 2940/2850 cm^−1^ bands (Figure S8C, Supporting Information). We extensively washed the cleared brains and slices in PBS and employed the unique peak of the solvent (≈3060 cm^−1^) to assess its clearance. Indeed, prolonged washing caused a significant reduction in the 3060 cm^−1^ peak, but the spectra remained marred with the solvents’ signatures. Nevertheless, in both ECi‐ and uDISCO‐brain slices, the lipid band was significantly reduced, indicating reduction in lipids compared to proteins (Figure S8D,E, Supporting Information). As a control, we used an additional clearing method, Sca*l*e, owing to the fact that its hydrophilic clearing reagents do not overlap with 2940/2850 cm^−1^ band.^[^ ^37^ ^]^ RS analysis revealed that Sca*l*e preserves lipids, as previsouly suggested (Figure S8F, Supporting Information).^[^ ^39^ ^]^

**Figure 4:**
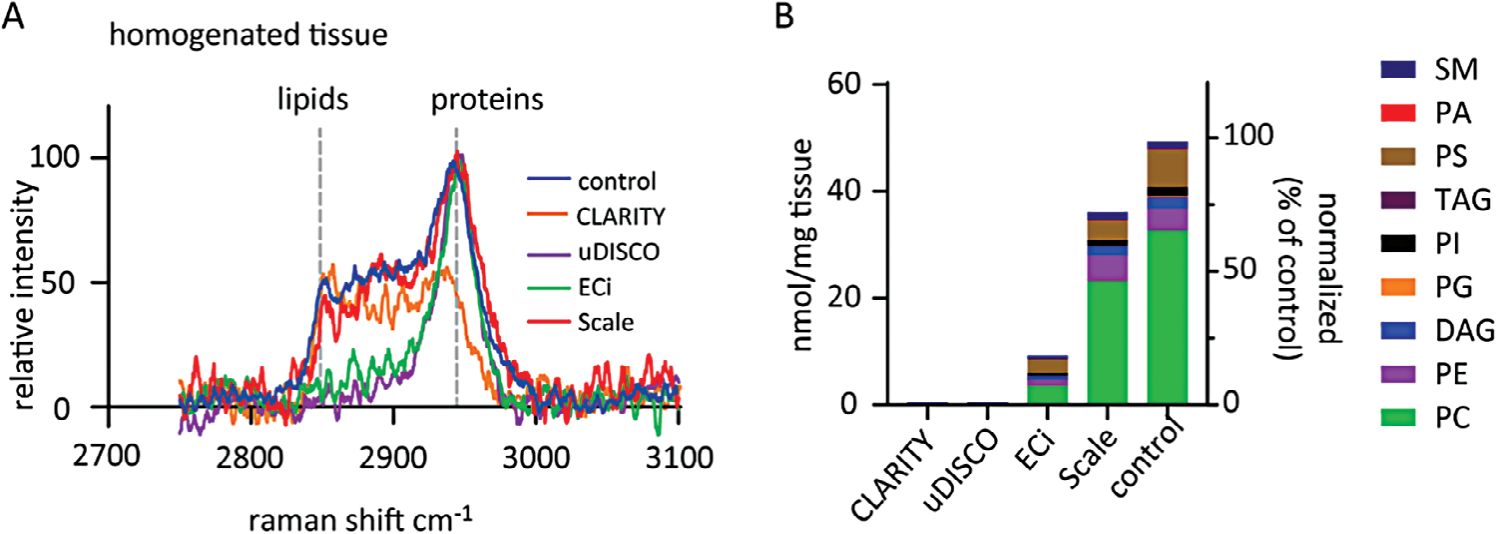
Quantitation of lipid content in cleared brains via Raman spectroscopy and Lipidomics. A) High wavenumber region of the Raman spectrum from non‐treated (control) and cleared brain tissue homogenates. ECi and uDISCO samples were extensively washed in PBS before homogenization. Relevant peaks for protein and lipid content are noted by dashed line. B) Lipidomics analysis of cleared brain samples. Lipid amount and relative composition measured in non‐treated (control) and cleared brain samples (from 150 µg homogenated samples) by mass spectrometry. PA – phosphatidate, PC – phosphatidylcholine, PE – phosphatidylethanolamine, PG – phosphatidylglycerol, PI – phosphatidylinositol, PS – phosphatidylserine, DAG – diacylglycerol, TAG – triacylglycerol, SM – sphingomyelin. Note the complete loss of lipids from CLARITY and uDISCO samples, closely followed by ECi. Sca*l*e samples retain most lipids.

Next, to better compare the ratios between different clearing techniques (and bypass potential differences that may arise from acquisition at varying regions), we homogenized the cleared‐brains. Homogenates from PBS washed ECi‐ and uDISCO‐brains showed a sharp attenuation in the 2850 cm^−1^ band, representing a strong decrease of lipids in the samples (**Figure** **4**A, magenta and green), compared to controls (Figure 4A, blue). Sca*l*e‐homogenates did not show any reduction in the protein/lipid ratio (Figure 4A, red). However—and interestingly—CLARITY homogenates, similarly to CLARITY‐brain slices, displayed a 2940/2850 cm^−1^ ratio of ≈1 indicating a relatively larger reduction in proteins rather than lipids (Figure 4A, orange). Thus, our RS measurements indicate a strong reduction in lipids following ECi and uDISCO‐clearing, but not following Sca*l*e. It also suggests that CLARITY is de‐proteinating as it is delipidating, which could explain the partial restoration in MRI‐contrast of CLARITY‐brains (Figure 3A), as ratio between “endogenous contrast‐agents” (i.e., proteins) and water is an essential factor for MRI‐contrast.^[^ ^23^, ^26^ ^]^ However, in our hands RS did not provide a conclusive assessment of lipid‐content in cleared brains owing to the relative‐nature of the method and, importantly, due to the overlapping spectra of solvents and lipids.

### 2.6 Quantitative Lipidomics of Cleared Brains by Mass Spectrometry

To accurately quantitate the extent of delipidation, we reverted to mass‐spectrometry (MS) lipidomics. Notably, despite the method's precision, resolution and common usage, it has not been previously employed to examine cleared specimens. We analyzed control and cleared homogenized mouse brains in duplicates of 150 and 300 µg per sample (Figure S9, Supporting Information). Control samples (PFA‐fixed) yielded 53 nmol lipids/mg tissue, highly consistent with previous reports. CLARITY and uDISCO‐samples exhibited near complete loss of all lipids in the brains (0.41 and 0.71 nmol mg^−1^ lipids, respectively), demonstrating 99.2% and 98.7% delipidation, respectively. ECi‐samples showed slightly higher remaining lipid content, 9.9 nmol mg^−1^ lipids, representing 81% delipidation (Figure 4B; Figure S9, Supporting Information). Lastly, Sca*l*e‐samples contained, on average, 38 nmol mg^−1^ lipids, thereby demonstrating that the method preserves most lipids in the tissue (i.e., retains >70% lipids) and is thereby very poorly delipidating (≈30% delipidating). Both Sca*l*e and Eci displayed the same lipid composition as in controls, with a remaining and high proportion of phosphatidylcholine (PC), phosphatidylethanolamine (PE), and phosphatidylserine (PS) (Figure 4B; Figure S9B, Supporting Information). Thus, our lipidomics analysis firmly demonstrates that CLARITY and uDISCO are equally—and potently—delipidating, closely followed by ECi, whereas Sca*l*e preserves most of the lipids in the tissue. Thus, the MRI‐contrast observed in CLARITY‐ and uDISCO‐brains, as well as in ECi‐brains, cannot be attributed to remaining lipids in the tissue. We therefore conclude that delipidation does not prevent MRI‐contrast in brains cleared by, at least, three different clearing methods.

### 2.7 Loss of MRI‐Contrast in Sca*l*e‐Treated Brains

**Figure 5:**
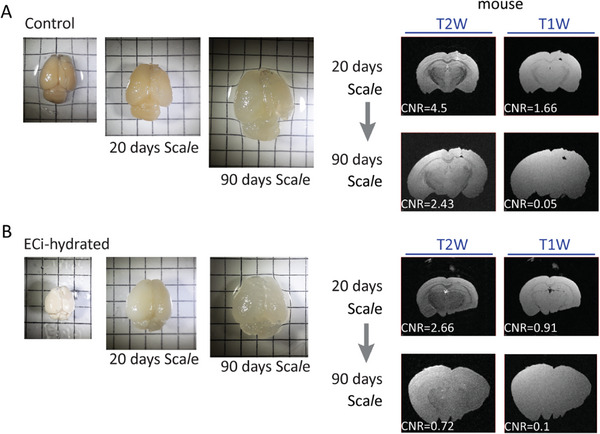
Progressive Sca*l*e treatment reduces MRI‐contrast. Control (A) and hydrated‐ECi‐cleared (B) mouse brains were imaged on 0.5‐cm grid paper, before and following incubation in Sca*l*e‐clearing reagents for 20 and 90 days (left). Coronal T1W and T2W MRI and CNR values are shown. CNR is calculated as in Figure 2C.

Having recognized that Sca*l*e‐clearing does not eliminate the vast majority of lipids from brain tissues (Figure 4), we deemed it as an added control for evaluating the impact of lipids on ex vivo MRI‐contrast. In fact, we view Sca*l*e as a more suitable control for CLARITY, as both methods involve hyperhydration (as opposed to profound dehydration by solvent‐based methods, such as uDISCO and ECi), while differing in the methods’ extent of delipidation. We cleared brains by Sca*l*e, and MRI imaged them following 20 and 90 days, representing partial and full clearing of the tissue, respectively. This treatment gradually cleared the brains, which appeared gelatinous and significantly expanded, as reported earlier (**Figure** **5**; Figure S10A, Supporting Information).^[^ ^39^ ^]^ Of note, these traits are highly reminiscent of CLARITY‐brains (see Figure 1). Unexpectedly, and in spite of retaining ≈70% of lipids in the tissue (see above), Sca*l*e‐cleared brains displayed very poor MRI‐contrast, much like CLARITY, especially past 90 days of treatment (Figure 5; Figure S10B, Supporting Information). This observation excludes lipids as the major source for ex vivo MRI‐contrast; however, it highlights hyperhydration and tissue expansion as potential culprits for the loss of MRI‐contrast.

To directly examine the role of tissue expansion for MRI‐contrast, we decided to apply the reagents used in the Sca*l*e process onto uDISCO and ECi‐treated brains (i.e., that have already been extensively delipidated). Sca*l*e‐treatment of uDISCO‐ and ECi‐brains instigated similar changes in color and consistency to the already cleared brains and, importantly, prompted strong expansion of the brains (Figure 5; Figure S10, Supporting Information). Notably, this double clearing caused robust increase in the MRI‐signal (hyperhydration) and concomitant reduction in MRI‐contrast; T1W showing higher susceptibility to loss in MRI‐contrast (Figure 5B; Figure S10B, Supporting Information). Together, Sca*l*e‐treated brains lack MRI‐contrast despite presence of lipids in samples. Sca*l*e‐treated uDISCO‐ and ECi‐brains undergo expansion and hyperhydration—without further delipidation—and this effect is sufficient to abolish MRI‐contrast. Our results thereby underscore tissue expansion and hyperhydration of the tissue as the underlying mechanism for loss of MRI‐contrast in cleared specimen. In support, ETC‐treatment of control, ECi‐ and uDISCO‐cleared brains instigated similar effects on tissue size (i.e., expansion) and reduction in MRI‐contrast without changing the lipid content.^[^ ^33^ ^]^ To mimic this effect, we imaged water‐absorbing gel beads (composed of super‐absorbent polymers, akin to CLARITY's hydrogel) by MRI (Figure S11A, Supporting Information). When placed in water, the beads undergo isotropic expansion and rapid hyperhydration, without change in the compositions of the beads. We thereby placed them in water and immediately imaged by MRI, and noted that each bead produced a particularly strong T2W image (Figure S11B, Supporting Information). However, this signal progressively diminished as the beads expanded—ultimately losing all MRI‐contrast—and detectability (Figure S11C, Supporting Information). Thus, this further demonstrates the tight association between expansion by hyperhydration and loss of MRI‐contrast.

### 2.8 Multimodal Ex Vivo LSM‐MRI of Cleared Brains

**Figure 6:**
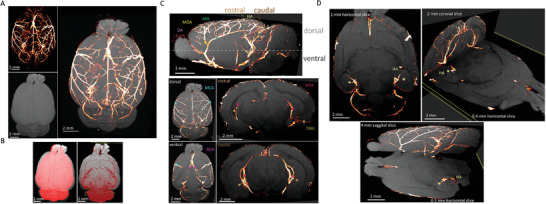
Multimodal ex vivo LSM‐MRI imaging of uDISCO‐cleared rat brain. A) Whole brain cerebral vasculature, fluorescently labeled in vivo by EB, was initially cleared by uDISCO, immersed in RI‐matching solution, and imaged by LSM (left, top). Samples were then hydrated and scanned by MRI (left, bottom). The acquired images were registered to a single 3D image (right). Horizontal maximal intensity projections are presented. B) Slight variations in size of samples during acquisition by the two imaging modalities. Surface filled projections left) and a single 0.4 mm horizontal slice (right) of the uDISCO cleared brains as acquired by LSM (red) and by MRI (right) following hydration. Note the slight expansion of the samples by hydration (gray borders). Thus, no corrections of the images (e.g., isotropic stretching of LSM images) were required for registration of images. C) Annotation of major blood vessels is enabled due to MRI landmarks. Registration of 4‐mm MR‐images with LSM images are shown (top‐sagittal; dorsal and ventral horizontal sections, middle and bottom panels, respectively). Coronal 0.6 mm slices in rostral and caudal positions are shown on the right. ACA‐anterior cerebral artery, MCA‐ middle cerebral artery. APA‐azygos pericallosal artery, MOA‐medial orbitofrontal artery, OA‐Olfactory artery, HA‐hippocampal arteries, LHIA‐longitudinal hippocampal arteries, TRHI‐ transverse hippocampal arteries. D) Annotation and orientation of hippocampal arteries (HA) seen in contrasting MRI and LSM micrographs, observed from various positons and slice thickness.

After establishing the necessary conditions to render uDISCO‐brains suitable for MRI, our goal was to leverage this capability to develop a multimodal ex vivo LM‐MRI imaging method. Notably, this has yet to be attempted, likely owing to the preconception that cleared specimen are simply incompatible with MRI. We then stained rat brain vasculature in vivo by Evans blue, a red fluorescent dye that predominantly marks major blood vessels.^[^ ^40^ ^]^ Then, brains were PFA‐fixed, extracted and cleared by uDISCO. We then first imaged the brain by LSM, followed by its hydration in PBS and imaging by high‐resolution MRI (**Figure** **6**). Ex vivo LSM‐MRI images could then be automatically registered by standard techniques, especially in light of the fact that re‐hydration of uDISCO‐brains did not cause major changes to the tissue's volume (Figure 6B and above). The mesoscale anatomical MRI images facilitated identification of major blood vessels across the brain at various depths and brain regions (Video S1, Supporting Information). Major veins and arteries, such as hippocampal arteries, could be accurately detected in the LSM image and correctly oriented within the hippocampal structure obtained by the correlated MRI image (Figure 6C). Visualization of the registered images, with both MRI‐landmarks and fluorescent details provided by LSM, offers a comprehensive look into the organization of the vessels within the context of the entire brain (i.e., at the mesoscale) (Figure 6D). It is worth noting that in our study, the co‐registration of the two imaging datasets required very minor adjustments, primarily involving small corrections for size (see Figure 6B). This is because we aligned MRI and light microscopy images of chemically‐cleared brains that have been acquired after the clearing process. Therefore, any deformations or alterations in the tissue that might have occurred during brain extraction from the skull and/or during the clearing process were equally reflected in both sets of images. This differs from previous studies where MRI images were specifically obtained before the clearing process (and even before brain extraction from the skull), whereas light microscopy imaging was carried‐out afterwards. In those instances, the imaged specimens varied significantly in size, volume, shape, and structure, making registrations more challenging (see, e.g., ref. [41]).

## 3 Discussion

One rapidly evolving field of study is the study of the brain in its entirety.^[^ ^42^ ^]^ A particularly powerful mesoscale imaging modality for this endeavor is MRI, especially when combined with microscale imaging modalities, for instance LM.^[^ ^43^, ^44^ ^]^ This combination mandates the presence of MRI‐contrast in the sample and its transparency for light to penetrate the sample. Nevertheless, prior to this report, the prevailing consensus maintained that optically‐cleared specimens, particularly those subjected to the CLARITY method, are deemed unfit for MRI. This has been attributed to absence of MRI‐contrast due to extensive tissue delipidation.^[^ ^2^, ^25^, ^26^, ^28^ ^]^ Nevertheless, this assumption runs counter to an extensive body of literature that underscores the potential contribution of other brain features and molecules to MRI‐contrast.^[^ ^3^, ^17^, ^18^, ^22^, ^45^ ^]^ It also fails to describe whether alternative clearing methods, including non‐delipidating approaches, produce similar outcomes and whether it is feasible to reestablish MRI‐contrast in cleared specimens. These issues underscore the limitations of the current *lipids‐to‐contrast* hypothesis (see introduction and^[^ ^2^, ^25^, ^26^, ^27^, ^44^ ^]^), and why the use of MRI‐LM multimodality is not under active developments.

Our initial goal was to conduct a thorough comparative analysis of different clearing methods concerning their compatibility with MRI. To our surprise, our explorations revealed that the popular uDISCO‐ and ECi‐clearing methods can be readily adapted to achieve MRI‐compatibility (by simple rehydration, Figure 2). This discovery prompted us to explore the possibility of restoring MRI‐contrast in CLARITY‐cleared brains, which we indeed achieved by slight dehydration and shrinkage of the samples, all without the need for re‐introducing lipids (Figure 3).

We then confirmed that the extant MRI‐contrast did not arise from partial delipidations of the tissues, by demonstrating that uDISCO and ECi remove 99% and 80% of all lipids, respectively, which are comparable to the extent of delipidation achieved by CLARITY (99%) (Figure 4). We further show the disconnect between lipid‐content and MRI‐contrast by imaging Sca*l*e‐cleared brains—Sca*l*e retains the majority of lipids in the tissue (≈30% delipidation), though Sca*l*e‐cleared brains do not exhibit MRI‐contrast (Figure 5).

**Figure 7:**
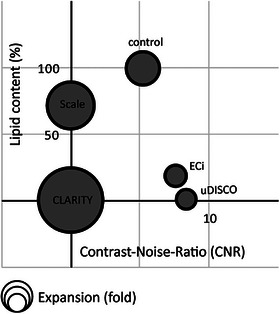
Tissue expansion, but not lipid content, is associated with loss of MRI‐contrast. A 3D plot showing the relationships between lipid content (Y‐axis), MRI‐contrast (CNR values, X‐axis) and change in volume (circle size) for the different tissue‐clearing approaches. Lipid content (%) is based on lipidomics data, CNR values are based on T1W MR images. Relative sample volume was assessed at the end of each tissue‐clearing treatment.

Both Sca*l*e‐ and CLARITY‐clearing methods produce cleared samples with similar characteristics, as both techniques are hyperhydrating and tissue‐expanding. These associated features were also observed when uDISCO or ECi‐brains were treated by ETC^[^ ^33^ ^]^ or by reagents used in Sca*l*e which reduced contrast (Figure 5; Figure S10, Supporting Information). This leads us to propose that hyperhydration and tissue expansion reduces ex vivo MRI‐contrast in chemically‐cleared brains, due to the strong and uniform MRI‐signal (**Figure** **7**). We also propose that in cases of complete delipidation, such as in CLARITY and uDISCO‐cleared brains, other tissue components, like proteins and iron retained in the cleared tissue, can provide significant sources MRI‐contrast (Figure S7, Supporting Information). However, this contrast is not apparent in CLARITY‐ and Sca*l*e‐brains because of the reduction in the effective concentrations of endogenous contrast agents due to dilution caused by the hyperhydration and expansion of the tissue. Extremely hyperhydrated and expanded samples indeed provide very strong and homogenous MRI signal (due to excessive water), overshadowing any potential changes in signal strength between regions in the tissue. Based on these collective findings, we were confident to achieve multimodal MRI‐LSM imaging.

### 3.1 Application of Multimodal Imaging of Cleared Brains by LM and Ex Vivo MRI

Multimodal‐imaging is widely employed to create comprehensive 3D structural and functional brain maps and atlases,^[^ ^46^ ^]^ as this method enables integration of different datasets from methods that span different scales, for instance the micro‐ to the mesoscale.^[^ ^47^ ^]^ Indeed, optically‐cleared specimens that can achieve cellular resolutions have been combined with multiple imaging approaches, such as X‐ray tomography, cryo‐EM, photoacoustic imaging, Raman scattering, and ion‐conductance microscopy, to name a few.^[^ ^48^, ^49^, ^50^, ^51^, ^52^ ^]^ In fact, there are several reports that describe attempts to combine cleared specimen with MRI, denoted magnetic resonance histology (MRH).^[^ ^53^ ^]^ Briefly, MRH involves MR‐imaging of fixed and intact brain tissues in situ (i.e., within the skull), followed by LSM‐imaging of the removed and cleared brain. Nonetheless, this method has several limitations, primarily complication in the alignment and registration of the images obtained by the two modalities. Explicitly, the brain imaged in situ is not the same sample that is then imaged by the LSM, due to potential tissue‐damages during the extraction of the brain from the skull and potential distortions in tissue size during its clearing. Thus, ex vivo MRI of cleared specimen has not been shown to date, owing to the limiting *lipid‐to‐contrast* hypothesis (see ref. [17] and [44])

Here, we present a method to bypass these limitations. We demonstrate the compatibility of optically‐cleared brains samples with MRI, in hope to pave the way for the development of intact tissue‐atlases based on multimodal MRI‐LSM datasets. We combine uDISCO‐clearing with MRI‐imaging as uDISCO‐clearing is relatively rapid, yields highly transparent cleared brains and induce sample shrinkage (which is ideal for imaging^[^ ^30^ ^]^). Furthermore, uDISCO samples do not undergo major changes in volume following the necessary hydration step for MR‐imaging and, importantly, uDISCO‐cleared brains exhibit a strong recovery of MRI‐contrast (Figures 2 and 6). Of note, hydration of uDISCO‐brains (which renders the tissue opaque and no longer compatible for LSM imaging) is reversible. In fact, hydration‐dehydration rounds are commonly employed for various processing of uDISCO samples (e.g., for antibody labeling), without noticeable impairment in tissue integrity.^[^ ^54^ ^]^ Thus, ex vivo LSM‐MRI imaging of a cleared specimen can be performed repeatedly, unlike MRH that can be performed only once.

We demonstrate ex vivo LSM‐MRI imaging of an entire uDISCO‐cleared brain and its major vascular network, achieving a resolution of <2 µm with LSM and 100 µm in‐plane resolution by ex vivo MRI scanning (Figure 6). The imaging process was notably efficient, with an LSM acquisition time of ≈40 min, T1W and T2W sequences by MRI taken within ≈20 min, and automatic co‐registration of 3D images accomplished within minutes. Notably, MRI facilitated the visualization and straightforward annotation of the vascular network based on the spatial context (Figure 6; Video S1, Supporting Information). Thus, we suggest that the method can be adapted for semi‐high throughput histopathology applications.

In conclusion, our study reveals that lipids are not the sole source of ex vivo MRI‐contrast. We have introduced straightforward protocols to make cleared‐brains compatible for MRI, and we have provided robust support for the mechanisms underlying the loss of MRI‐contrast. These findings have the potential to influence the development of new clearing techniques aimed at producing MRI‐compatible samples, and to motivate the development of MRI‐LSM atlases from various tissues. We also suggest that the presented multimodal ex vivo LSM‐MRI method should be ideal for the development of novel multimodal agents for MRI and LM, as our multimodal approach offers a unique means to rapidly validate MRI‐signals with fluorescent signals obtained from the same sample ex vivo.^[^ ^55^, ^56^ ^]^ Lastly, we envision that ex vivo LSM‐MRI could find applications in pharmacological research and clinical histological assessments, enabling the rapid and precise detection of pathologies in 3D and at various scales.^[^ ^27^, ^44^, ^57^ ^]^

## 4 Experimental Section

### Animals and Ethics

Three‐weeks to 6 months old, male and female, C57BL/6 mice and Sprague Dawley rats (Envigo, Israel) were used. All animal procedures were in accordance with the guidelines and regulations of the Technion and were approved by the Animal Care and Use Committee of the Technion – Israel Institute of Technology (Haifa, Israel, Ethic number IL‐121‐08‐19). Animals were transcardially perfused with phosphate‐buffered saline (PBS) (02‐023‐5A, Biological Industries) and neutral buffered 10% formalin (HT5011, Sigma–Aldrich). Fixed‐brains were removed and placed in formalin at 4 °C for 24 h, then washed and incubated in PBS at 4 °C until treated or imaged.

### Tissue‐Clearing Techniques

*Clarity* ^[^ ^31^ ^]^: The procedure was performed using a commercial CLARITY‐clearing system and reagents (X‐CLARITY; Logos Biosystems). Briefly, whole brains were infused with hydrogel monomers at 4 °C overnight (cat #: C1310X, Logos Biosystems) and a subsequent hydrogel polymerization step was performed at 37 °C for 3 h at −90 kPa (cat #: C20001, Logos Biosystems). Polymerized brains were then actively cleared in electrophoretic tissue‐ clearing (ETC) solution containing 200 mm boric acid and 4% (wt/vol) SDS (pH 8.5) (C13001, Logos Biosystems) by a tissue‐clearing system (cat #: C10001, Logos Biosystems). Unless otherwise mentioned, whole mouse brain were placed in the electrophoretic chamber under constant current of 1–1.2 A for 6–8 h, whereas whole rat brains at 1.2 A for 10–12 h.

*uDISCO* ^[^ ^30^ ^]^: Whole brains were dehydrated by sequential immersion in incrementing concentrations of *tert*‐butanol (30, 50, 70, 80, 90, 96, and 100% vol in DDW; cat #: 360 538, Sigma–Aldrich) for 4–12 h in each solution along gentle shaking at 34 °C. Then, samples were delipidated by incubation in Dichloromethane (DCM; cat #: 270 997, Sigma–Aldrich) for 1–2 h Finally, samples were incubated in a refractive index (RI)‐matching solution consisting of 1:2 benzyl alcohol (cat #:24 122, Sigma–Aldrich) and benzyl benzoate (cat#: B6630, Sigma–Aldrich), denoted BABB solution, supplemented with DPE (cat #: 67 334, Sigma–Aldrich) at 15:1 BABB to DPE ratio, and 0.4% vol DL‐alpha‐tocopherol (Vitamin E; cat #A17039, Alfa Aesar).

*ECi* ^[^ ^29^ ^]^: Whole brains were dehydrated by sequential immersion in incrementing concentrations of ethanol (30, 50, 70, 100% vol in DDW; 30–70% solutions were adjusted to pH 9) supplemented with 2% Tween‐20 (cat #: P1379, Sigma–Aldrich) for 12–24 h in each solution with gentle shaking at 4 °C. Then, samples were delipidated in 100% ethyl‐cinnamate (ECi, cat #: 112 372, Sigma–Aldrich) for at least 24 h, until desired transparency is obtained.

*Scale* ^[^ ^39^ ^]^: Whole brains were immersed in Sca*l*e‐A2 solution containing: 4 m urea (cat #: U5128, Sigma–Aldrich), 10% (v/v) glycerol (cat #: 191 612, Sigma–Aldrich) and 0.1% (v/v) Triton X‐100 (cat #: X100, Sigma‐Aldrich) adjusted to pH 7.6, with gentle shaking at room temperature (RT). Whole brains were treated for up to 90 days.

### Secondary Treatments of Cleared Brains

Hydration of ECi and uDISCO ‐cleared brains was achieved by immersion of the entire samples in PBS at RT for 3 to 30 days. CLARITY‐ brains were immersed in 100% 2,2′‐thiodiethanol (TDE) at RT for 5–7 days (full treatment) or overnight (partial treatment). After TDE incubation, CLARITY brains were immersed in PBS with and without 5 mm gadolinium (Gd^3+^)‐based contrast‐agent (GBCA) (Dotarem, Guerbet) at RT 7 days. For contrast agent injection (Figure S5, Supporting Information), 1 µl of saline or 0.5 mm GBCA, was injected into one hemisphere of the cleared brain with a commercial microinjector (NanoJectIII, Drummond Scientific) using glass capillaries (cat #: 504 949, WPI).

### Imaging of Brains and Assessment of Dimension Changes

Micrographs of brains, placed on top of an empty 10 cm diameter round dishes (e.g., Figure 2A; Figure S7, Supporting Information) and inside refractive index (RI)‐matching solution (Figure 1B), were taken by the Fusion FX7 imaging system (Edge Spectra, VILBER). Snapshots of brains placed on top of a grid paper, without any immersion, were taken using a regular camera. Images were taken before the samples were scanned by MRI. The expansion/shrinkage assessment was done on the 2D area of the same brains after different treatments (Figure 2A) by the number of pixels (ImageJ, NIH).

### Magnetic Resonance Imaging

Images were acquired using a 9.4T MRI system horizontal bore system (Bruker Biospec, Ettlingen, Germany) interface with Avance III console, equipped with a cylindrical transmit volume coil (86 mm inner diameter) and a surface coil (20 mm diameter) for detection. Brains were placed in cylindrical tubes and immersed in Fomblin (Fomblin‐Y LVAC 06/6, cat #: 317 926, Sigma–Aldrich) to minimize susceptibility artifacts. MRI protocols included coronal and horizontal anatomical T2W and T1W scans. T2W images were acquired using a Rapid Acquisition with Relaxation Enhancement sequence (RARE), at 0.4–0.6 mm slice thickness of 27–32 Slices, 100 µm in plane resolution with the following features: TR = 3500–4110 ms, TE = 36 ms, RARE factor = 12, FOV = 1.6 × 1.6 cm^2^ or 1.92 × 1.92/2.2 cm^2^, matrix size = 160 × 160 or 192 × 192/220, number of averages = 6. T1W images were acquired with a fast low angle shot (FLASH) sequence with the same geometry and resolution as T2W sequences, and with TR/TE = 320/4 ms, 30° pulse, number of averages = 6. For brains with injected GBCA (Figure 4), an additional protocol of 3D T1W FLASH images were acquired, with the same geometry and resolution as before, and with TR/TE = 15/4.4 ms, 15° pulse, number of averages = 2. For co‐registration with light microscopy, T1W MR‐images of hydrated uDISCO‐cleared brains were acquired using FLASH sequence TR/TE = 362/4 ms, 30° pulse at 100 × 100 × 210 µm^3^ resolution, and number of averages = 10. Data processing was performed using Medical Image Processing Analysis, and Visualization (MIPAV) software (NIH). Regions of interest (ROIs) were manually selected and traced. The MRI‐contrast was calculated as contrast‐to‐noise ratio (CNR) of the signal intensity difference between cortex (or hippocampus) and the corpus callosum, divided by the background (i.e., noise). Intensity profiles were obtained by tracing a horizontal line across grayscale images and plotted as intensity of voxel of interest (VOI) along the trajectory of the line.

### Quantitative MRI

T1, T2 and T2* measurements were performed on coronal MRI images with 100 µm in plane resolution and slice thickness of 0.4 mm, FOV = 1.6 × 1.6 cm^2^ and matrix size = 160 × 160. Multi slice multi echo (MSME) sequence was used for T2 mapping, with TR = 2500 ms, and 10 echo times: TE 10–100 ms, number of averages = 3, scan time 15 min. RARE sequence with variable TR values was used for T1 mapping TE 24.33, and 12 TRs: 70–8000 ms, number of averages = 2, scan time 14 min. For T2* mapping, multi gradient echo (MGE) sequence was used with TR = 200 ms, 12 echo times TE = 3–35. Number of averages = 8, scan time 3.5 min. MRI maps were generated using a custom build software in Matlab (Matworks, MA) by performing exponential pixel‐wise curve fitting. Relaxations times were measured in selected ROI (manually annotated).

### Brain Tissue Homogenization

PFA‐fixed mouse brains were weighed and optically cleared. ECi and uDISCO‐cleared brains were extensively washed with PBS for 72 h prior homogenization. To accelerate tissue clearing via Sca*l*e, the brain was sectioned to large pieces (2–3 mm thick) to facilitate the diffusion of the reagents into the entire brain (for 2 weeks). Samples were homogenized using a tissue homogenizer.

### Mass‐Spectrometry Lipidomics

Two 30 µl samples from each brain homogenate (at 5 and 10 mg/ml) were sent to a commercial lipidomic analysis (Lipotype GmbH, Germany). Briefly, lipid extraction was performed by using a two‐step chloroform/methanol procedure. Samples were spiked with lipid class‐specific internal standards prior to extraction. After extraction, the organic phase was dried and resuspended in mass‐spectrometry acquisition mixture. The lipid extracts were subjected to MS analysis, as previously described.^[^ ^58^, ^59^, ^60^, ^61^ ^]^

### Raman Spectroscopy

Raman spectra were acquired using an upright Confocal micro‐Raman Microscope (LabRAM HR evolution, HORIBA scientific). Samples were illuminated by 532 nm laser (100% laser intensity) through ×50 objective (LWD). The acquisition time was 10 s/point (except the spectrum shown in Figure S8A, Supporting Information, acquired at 100 s/point) at three accumulations. Samples (brain homogenates at 100 mg mL^−1^), thick tissue slices or different clearing solutions and reagents were placed and sampled on quartz slides (cat #: 04 2295, Alfa Aesar). The same CLARITY‐cleared sample was analyzed at the different ETC time points (3 and 9 h).

### Measuring Iron Content Using Inductively Coupled Plasma‐Optical Emission Spectrometry (ICP‐OES)

PFA‐fixed brains were cut in the midline and each hemisphere was weighed and treated by a different tissue‐clearing protocol (or a non‐cleared control). Samples were gently washed by PBS then singed at 550 °C for 5 h, and remaining ashes were dissolved in 1% nitric acid. The dissolved samples were filtered (0.45 µm syringe filters), and analyzed using inductively coupled plasma‐optical emission spectrometry (ICP‐OES) (PlasmaQuant 9000 elite Analytik Jena, Germany).^[^ ^56^ ^]^ Multiple standards (0.01, 0.1, 1, 10, 100 ppm) were used for calibration. Brain samples contained >0.1 ppm iron per sample, whereas 0.5 mL solutions of PBS, ECi, and uDISCO RI‐matching solution had less than trace amounts of iron (<0.005 ppm).

### Labeling of Brain Vasculature

We employed the Evans blue dye (EB; E2129, Sigma–Aldrich) to stain brain major vasculature, as commonly reported.^[^ ^40^ ^]^ Briefly, EB (2%, 150 µl) was injected intraperitoneally to rats, 12 h before they were transcardially perfused, and brains extracted.

### Light‐Sheet Microscope Image Acquisition and Post‐Processing

Brains were imaged by a commercial LSM (SmartSPIM, LifeCanvas). Cleared brains were mounted on a handle using super‐glue and placed for overnight equilibration inside a chamber filled with uDISCO RI‐matching solution (see section 2.2.2). Red fluorescence of EB was obtained by 648 nm excitation and emission was collected over the range of 680/42 nm. The system is equipped with dual illumination objectives (NA 0.20). Images were acquired using a ×3.6 objective NA 0.2/WD 17 mm (TL4X‐SAP, Thorlabs), at 2 ms exposure time and immersion RI 1.56. The spatial resolution was 1.8 µm (X‐Y axis), at 2 µm steps (Z axis). A sCMOS camera system with 2048 × 2048 pixel resolution acquired images at 20 FPS (Hamamatsu ORCA Flash 4.0 V3). Images were de‐striped and stitched (10% overlap) using open‐source codes (Pystripe and TeraStitcher, respectively).

### Analysis and Co‐Registration of MRI and LSM 3D Datasets

Both MRI and LSM datasets were brought to a common resolution. A 25 µm cubic resolution was chosen as target to maintain the high resolution of the LM images, and for matching with standard brain atlases (Allen atlas). Thus, MRI scans at 100 × 100 × 210 µm pixel resolution were interpolated, whereas LSM data sets acquired at at 1.8 × 1.8 × 2 µm resolution were down sampled. Next, co‐registration was obtained using the python‐based *Elastix* toolbox. The LSM dataset was set as reference image, and MRI dataset was aligned to it. Briefly, co‐registration entails an initial rigid body affine transformation, followed by a fine, non‐linear (B‐Spline) optimization. This method allows accounting for small deformations in brain morphology. Registered LSM and MRI volumes were converted to *.ims format for 3D visualization in IMARIS (Bitplane, v9.2.1). Background subtraction tool was used before LSM maximum intensity projection.

### Statistical Analysis

Data are presented as mean ± SEM, unless otherwise mentioned (e.g., Table S1, Supporting Information). Statistics were obtained by Prism (8.4.0, GraphPad). Multiple group comparison was performed using one‐way ANOVA followed by Sidak *post hoc* test.

## Conflict of Interest

The authors declare no conflict of interest.

## Author Contributions

S.O. and G.S. contributed equally to this work. S.B. and S.O. designed the study, interpreted results, and wrote the paper. S.O. and G.S. performed and analyzed the experiments. S.O. and R.H. performed preliminary experiments. M.O.K. contributed to discussion. All authors wrote and approved the final version of the manuscript.

## Supporting information

Supporting Information

Supplemental Video 1

## Data Availability

The data that support the findings of this study are available from the corresponding author upon reasonable request.

## References

[1] M. Symms , J. Neurol. Neurosurg. Psychiatry 2004, 75, 1235. 15314108 10.1136/jnnp.2003.032714 PMC1739217

[2] C. Leuze , M. Aswendt , E. Ferenczi , C. W. Liu , B. Hsueh , M. Goubran , Q. Tian , G. Steinberg , M. M. Zeineh , K. Deisseroth , J. A. McNab , NeuroImage 2017, 156, 412. 28411157 10.1016/j.neuroimage.2017.04.021 PMC5548623

[3] C. Stüber , M. Morawski , A. Schäfer , C. Labadie , M. Wähnert , C. Leuze , M. Streicher , N. Barapatre , K. Reimann , S. Geyer , D. Spemann , R. Turner , NeuroImage 2014, 93, 95. 24607447 10.1016/j.neuroimage.2014.02.026

[4] C. Birkl , J. Doucette , M. Fan , E. Hernández‐Torres , A. Rauscher , Magn. Reson. Med. 2021, 85, 2221. 33017486 10.1002/mrm.28543 PMC7821018

[5] N. Petridou , S. J. Wharton , A. Lotfipour , P. Gowland , R. Bowtell , NeuroImage 2010, 50, 491. 20026280 10.1016/j.neuroimage.2009.12.052

[6] N. Yamada , S. Imakita , T. Sakuma , M. Takamiya , Radiology 1996, 198, 171. 8539373 10.1148/radiology.198.1.8539373

[7] O. Shtangel , A. A. Mezer , NMR Biomed 2020, 33, e4209. 31899589 10.1002/nbm.4209

[8] J. Luo , X. He , D. A. d'Avignon , J. J. H. Ackerman , D. A. Yablonskiy , J. Magn. Reson. San Diego Calif 2010, 202, 102. 10.1016/j.jmr.2009.10.005 PMC2818297 19879785

[9] J. H. Duyn , J. Schenck , NMR Biomed 2017, 30, e3546. 10.1002/nbm.3546 PMC5131875 27240118

[10] S. Eickhoff , N. B. Walters , A. Schleicher , J. Kril , G. F. Egan , K. Zilles , J. D. G. Watson , K. Amunts , Hum. Brain Mapp. 2005, 24, 206. 15543596 10.1002/hbm.20082 PMC6871702

[11] S. Filo , O. Shtangel , N. Salamon , A. Kol , B. Weisinger , S. Shifman , A. A. Mezer , Nat. Commun. 2019, 10, 3403. 31363094 10.1038/s41467-019-11319-1 PMC6667463

[12] S. H. Koenig , R. D. Brown , M. Spiller , N. Lundbom , Magn. Reson. Med. 1990, 14, 482. 2355830 10.1002/mrm.1910140306

[13] A. Mezer , J. D. Yeatman , N. Stikov , K. N. Kay , N.‐J. Cho , R. F. Dougherty , M. L. Perry , J. Parvizi , L. H. Hua , K. Butts‐Pauly , B. A. Wandell , Nat. Med. 2013, 19, 1667. 24185694 10.1038/nm.3390 PMC3855886

[14] H. E. Möller , L. Bossoni , J. R. Connor , R. R. Crichton , M. D. Does , R. J. Ward , L. Zecca , F. A. Zucca , I. Ronen , Trends Neurosci 2019, 42, 384. 31047721 10.1016/j.tins.2019.03.009

[15] J. D. Thiessen , Y. Zhang , H. Zhang , L. Wang , R. Buist , M. R. Del Bigio , J. Kong , X.‐M. Li , M. Martin , NMR Biomed 2013, 26, 1562. 23943390 10.1002/nbm.2992

[16] N. B. Walters , S. B. Eickhoff , A. Schleicher , K. Zilles , K. Amunts , G. F. Egan , J. D. Watson , Hum Brain Mapp. 2007, 28, 1. 16773636 10.1002/hbm.20267 PMC6871284

[17] A. De Barros , G. Arribarat , J. Combis , P. Chaynes , P. Péran , Front. Neuroanat. 2019, 13. 10.3389/fnana.2019.00068 PMC6616088 31333421

[18] B. Drayer , P. Burger , R. Darwin , S. Riederer , R. Herfkens , G. Johnson , AJR Am. J. Roentgenol. 1986, 147, 103. 3487201 10.2214/ajr.147.1.103

[19] R. G. Steen , W. E. Reddick , R. J. Ogg , Magn. Reson. Imaging 2000, 18, 361. 10788712 10.1016/s0730-725x(00)00123-5

[20] W. D. Rooney , G. Johnson , X. Li , E. R. Cohen , S.‐G. Kim , K. Ugurbil , C. S. Springer , Magn. Reson. Med. 2007, 57, 308. 17260370 10.1002/mrm.21122

[21] T.‐Q. Li , B. Yao , P. van Gelderen , H. Merkle , S. Dodd , L. Talagala , A. P. Koretsky , J. Duyn , Magn. Reson. Med. 2009, 62, 1652. 19859939 10.1002/mrm.22156 PMC3508464

[22] H.‐G. Shin , J. Lee , Y. H. Yun , S. H. Yoo , J. Jang , S.‐H. Oh , Y. Nam , S. Jung , S. Kim , M. Fukunaga , W. Kim , H. J. Choi , J. Lee , NeuroImage 2021, 240, 118371. 34242783 10.1016/j.neuroimage.2021.118371

[23] D. S. Richardson , J. W. Lichtman , Cell 2015, 162, 246. 26186186 10.1016/j.cell.2015.06.067 PMC4537058

[24] S. Daetwyler , R. P. Fiolka , Commun. Biol. 2023, 6, 502. 37161000 10.1038/s42003-023-04857-4 PMC10169780

[25] K. Baek , S. Jung , J. Lee , E. Min , W. Jung , H. Cho , Sci. Rep. 2019, 9, 2923. 30814611 10.1038/s41598-019-39634-z PMC6393517

[26] K. Lee , H. M. Lai , M. H. Soerensen , E. S.‐K. Hui , V. W.‐S. Ma , W. C.‐S. Cho , Y.‐S. Ho , R. C.‐C. Chang , Neuropathol. Appl. Neurobiol. 2021, 47, 441. 33107057 10.1111/nan.12673 PMC8048831

[27] S. Zhao , M. I. Todorov , R. Cai , R. A. Maskari , H. Steinke , E. Kemter , H. Mai , Z. Rong , M. Warmer , K. Stanic , O. Schoppe , J. C. Paetzold , B. Gesierich , M. N. Wong , T. B. Huber , M. Duering , O. T. Bruns , B. Menze , J. Lipfert , V. G. Puelles , E. Wolf , I. Bechmann , A. Ertürk , Cell 2020, 180, 796. 32059778 10.1016/j.cell.2020.01.030 PMC7557154

[28] N. Weiskopf , L. J. Edwards , G. Helms , S. Mohammadi , E. Kirilina , Nat. Rev. Phys. 2021, 3, 570.

[29] A. Klingberg , A. Hasenberg , I. Ludwig‐Portugall , A. Medyukhina , L. Männ , A. Brenzel , D. R. Engel , M. T. Figge , C. Kurts , M. Gunzer , J. Am. Soc. Nephrol. 2017, 28, 452. 27487796 10.1681/ASN.2016020232 PMC5280021

[30] C. Pan , R. Cai , F. P. Quacquarelli , A. Ghasemigharagoz , A. Lourbopoulos , P. Matryba , N. Plesnila , M. Dichgans , F. Hellal , A. Ertürk , Nat. Methods 2016, 13, 859. 27548807 10.1038/nmeth.3964

[31] K. Chung , J. Wallace , S.‐Y. Kim , S. Kalyanasundaram , A. S. Andalman , T. J. Davidson , J. J. Mirzabekov , K. A. Zalocusky , J. Mattis , A. K. Denisin , S. Pak , H. Bernstein , C. Ramakrishnan , L. Grosenick , V. Gradinaru , K. Deisseroth , Nature 2013, 497, 332. 23575631 10.1038/nature12107 PMC4092167

[32] K. Chung , K. Deisseroth , Nat. Methods 2013, 10, 508. 23722210 10.1038/nmeth.2481

[33] S. Oz , G. Saar , S. Olszakier , R. Heinrich , M. O. Kompanets , S. Berlin , Data Brief. 2023, 52, 109795. 38146303 10.1016/j.dib.2023.109795 PMC10749242

[34] J. Choi , E. Lee , J. H. Kim , W. Sun , Exp. Neurobiol. 2019, 28, 436. 31308802 10.5607/en.2019.28.3.436 PMC6614074

[35] J. H. Kim , M. J. Jang , J. Choi , E. Lee , K. D. Song , J. Cho , K. T. Kim , H. J. Cha , W. Sun , Sci. Rep. 2018, 8, 12815. 30143733 10.1038/s41598-018-31153-7 PMC6109102

[36] J. Li , P. Lin , Y. Tan , J.‐X. Cheng , Biomed. Opt. Express 2019, 10, 4329. 31453014 10.1364/BOE.10.004329 PMC6701556

[37] M. Wei , L. Shi , Y. Shen , Z. Zhao , A. Guzman , L. J. Kaufman , L. Wei , W. Min , Proc. Natl. Acad. Sci. U. S. A. 2019, 116, 6608. 30872474 10.1073/pnas.1813044116 PMC6452712

[38] K. Czamara , K. Majzner , M. Z. Pacia , K. Kochan , A. Kaczor , M. Baranska , J. Raman Spectrosc. 2015, 46, 4.

[39] H. Hama , H. Kurokawa , H. Kawano , R. Ando , T. Shimogori , H. Noda , K. Fukami , A. Sakaue‐Sawano , A. Miyawaki , Nat. Neurosci. 2011, 14, 1481. 21878933 10.1038/nn.2928

[40] M. I. Todorov , J. C. Paetzold , O. Schoppe , G. Tetteh , S. Shit , V. Efremov , K. Todorov‐Völgyi , M. Düring , M. Dichgans , M. Piraud , B. Menze , A. Ertürk , Nat. Methods 2020, 17, 442. 32161395 10.1038/s41592-020-0792-1 PMC7591801

[41] G. A. Johnson , Y. Tian , D. G. Ashbrook , G. P. Cofer , J. J. Cook , J. C. Gee , A. Hall , K. Hornburg , C. C. Kaczorowski , Y. Qi , F.‐C. Yeh , N. Wang , L. E. White , R. W. Williams , Proc. Natl. Acad. Sci. U. S. A. 2023, 120, e2218617120. 37068254 10.1073/pnas.2218617120 PMC10151475

[42] J. Alexander Bae , M. Baptiste , C. A. Bishop , A. L. Bodor , D. Brittain , J. Buchanan , D. J. Bumbarger , M. A. Castro , B. Celii , E. Cobos , F. Collman , N. M. Costa , S. Dorkenwald , L. Elabbady , P. G. Fahey , T. Fliss , E. Froudarakis , J. Gager , C. Gamlin , W. Gray‐Roncal , A. Halageri , J. Hebditch , Z. Jia , E. Joyce , J. Joyce , C. Jordan , D. Kapner , N. Kemnitz , S. Kinn , L. M. Kitchell , et al., bioRxiv 2023. 10.1101/2021.07.28.454025

[43] M. Y. Khodanovich , T. V. Anan'ina , E. P. Krutenkova , A. E. Akulov , M. S. Kudabaeva , M. V. Svetlik , Y. A. Tumentceva , M. M. Shadrina , A. V. Naumova , Biomedicines 2022, 10, 1556. 35884861 10.3390/biomedicines10071556 PMC9313422

[44] M. Morawski , E. Kirilina , N. Scherf , C. Jäger , K. Reimann , R. Trampel , F. Gavriilidis , S. Geyer , B. Biedermann , T. Arendt , N. Weiskopf , NeuroImage 2018, 182, 417. 29196268 10.1016/j.neuroimage.2017.11.060 PMC6189522

[45] C. Langkammer , N. Krebs , W. Goessler , E. Scheurer , K. Yen , F. Fazekas , S. Ropele , Neuroimage 2012, 59, 1413. 21893208 10.1016/j.neuroimage.2011.08.045 PMC3236994

[46] K. Amunts , C. Lepage , L. Borgeat , H. Mohlberg , T. Dickscheid , M.‐É. Rousseau , S. Bludau , P.‐L. Bazin , L. B. Lewis , A.‐M. Oros‐Peusquens , N. J. Shah , T. Lippert , K. Zilles , A. C. Evans , Science 2013, 340, 1472. 23788795 10.1126/science.1235381

[47] C. Bosch , T. Ackels , A. Pacureanu , Y. Zhang , C. J. Peddie , M. Berning , N. Rzepka , M.‐C. Zdora , I. Whiteley , M. Storm , A. Bonnin , C. Rau , T. Margrie , L. Collinson , A. T. Schaefer , Nat. Commun. 2022, 13, 2923. 35614048 10.1038/s41467-022-30199-6 PMC9132960

[48] M. Chourrout , H. Rositi , E. Ong , V. Hubert , A. Paccalet , L. Foucault , A. Autret , B. Fayard , C. Olivier , R. Bolbos , F. Peyrin , C. Crola‐da‐Silva , D. Meyronet , O. Raineteau , H. Elleaume , E. Brun , F. Chauveau , M. Wiart , Biomed. Opt. Express 2022, 13, 1620. 35415001 10.1364/BOE.438832 PMC8973191

[49] L. Li , J. Xia , G. Li , A. Garcia‐Uribe , Q. Sheng , M. A. Anastasio , L. V. Wang , Neurophotonics 2016, 3, 1. 10.1117/1.NPh.3.3.035001 PMC5696384 29181425

[50] V. Navikas , S. M. Leitao , K. S. Grussmayer , A. Descloux , B. Drake , K. Yserentant , P. Werther , D.‐P. Herten , R. Wombacher , A. Radenovic , G. E. Fantner , Nat. Commun. 2021, 12, 4565. 34315910 10.1038/s41467-021-24901-3 PMC8316521

[51] M. W. Tuijtel , A. J. Koster , S. Jakobs , F. G. A. Faas , T. H. Sharp , Sci. Rep. 2019, 9, 1369. 30718653 10.1038/s41598-018-37728-8 PMC6362030

[52] Y. Zhao , O. Bucur , H. Irshad , F. Chen , A. Weins , A. L. Stancu , E.‐Y. Oh , M. DiStasio , V. Torous , B. Glass , I. E. Stillman , S. J. Schnitt , A. H. Beck , E. S. Boyden , Nat. Biotechnol. 2017, 35, 757. 28714966 10.1038/nbt.3892 PMC5548617

[53] G. A. Johnson , Y. Tian , D. G. Ashbrook , G. P. Cofer , J. J. Cook , J. C. Gee , A. Hall , K. Hornburg , C. C. Kaczorowski , Y. Qi , F.‐C. Yeh , N. Wang , L. E. White , R. W. Williams , Proc. Natl. Acad. Sci. U. S. A. 2023, 120, e2218617120. 37068254 10.1073/pnas.2218617120 PMC10151475

[54] N. Renier , Z. Wu , D. J. Simon , J. Yang , P. Ariel , M. Tessier‐Lavigne , Cell 2014, 159, 896. 25417164 10.1016/j.cell.2014.10.010

[55] L. Amirav , S. Berlin , S. Olszakier , S. K. Pahari , I. Kahn , Front Neurosci. 2019, 13, 12. 30778281 10.3389/fnins.2019.00012 PMC6369355

[56] E. Thankarajan , S. Oz , A. Saady , K. Kulbitski , M. O. Kompanets , M. S. Eisen , S. Berlin , ChemBioChem 2023, 24, 202300172. 10.1002/cbic.202300172 37092744

[57] K. Schregel , L. Heinz , J. Hunger , C. Pan , J. Bode , M. Fischer , V. Sturm , V. Venkataramani , K. Karimian‐Jazi , D. A. Agardy , Y. Streibel , R. Zerelles , W. Wick , S. Heiland , T. Bunse , B. Tews , M. Platten , F. Winkler , M. Bendszus , M. O. Breckwoldt , J. Neurosci. 2023, 43, 5574. 37429718 10.1523/JNEUROSCI.1470-22.2023 PMC10376935

[58] D. Fitzner , J. M. Bader , H. Penkert , C. G. Bergner , M. Su , M.‐T. Weil , M. A. Surma , M. Mann , C. Klose , M. Simons , Cell Rep. 2020, 32, 108132. 32937123 10.1016/j.celrep.2020.108132

[59] R. Herzog , D. Schwudke , K. Schuhmann , J. L. Sampaio , S. R. Bornstein , M. Schroeder , A. Shevchenko , Genome Biol. 2011, 12, R8. 21247462 10.1186/gb-2011-12-1-r8 PMC3091306

[60] J. L. Sampaio , M. J. Gerl , C. Klose , C. S. Ejsing , H. Beug , K. Simons , A. Shevchenko , Proc. Natl. Acad. Sci 2011, 108, 1903. 21245337 10.1073/pnas.1019267108 PMC3033259

[61] M. A. Surma , M. J. Gerl , R. Herzog , J. Helppi , K. Simons , C. Klose , Sci Rep. 2021, 11, 19364. 34588529 10.1038/s41598-021-98702-5 PMC8481471

