# Supplementary material for: Revealing the MRI‐Contrast in Optically Cleared Brains
Source: Adv Sci (Weinh). 2024 Apr 22;11(22):2400316. doi: 10.1002/advs.202400316 (PMC11165557; doi:10.1002/advs.202400316)
Supplement: Supplementary file 1 — Supporting Information [file ADVS-11-2400316-s001.pdf]

ADVANCED SCIENCE
Open Access

Supporting Information

for *Adv. Sci.*, DOI 10.1002/advs.202400316

Revealing the MRI-Contrast in Optically Cleared Brains

*Shimrit Oz, Galit Saar, Shunit Olszakier, Ronit Heinrich, Mykhail O. Kompanets and Shai Berlin**

Supporting Information for

Revealing the MRI-contrast in Optically Cleared Brains

Shimrit Oz[1], Galit Saar[2], Shunit Olszakier[1], Ronit Heinrich[1], Mykhail O. Kompanets[3], Shai Berlin[1]

1. Department of Neuroscience, Faculty of Medicine, Technion-Israel Institute of Technology, Haifa, Israel
2. Biomedical Core Facility, Faculty of Medicine, Technion-Israel Institute of Technology, Haifa, Israel
3. L.M. Litvinenko Institute of Physico-Organic Chemistry and Coal Chemistry, National Academy of Sciences of Ukraine, Kyiv, Ukraine.

Corresponding author: Dr. Shai Berlin, shai.berlin@technion.ac.il

This file includes

1. Figures S1 to S11
2. Table S1

OZ et al. Supplementary Figure 1

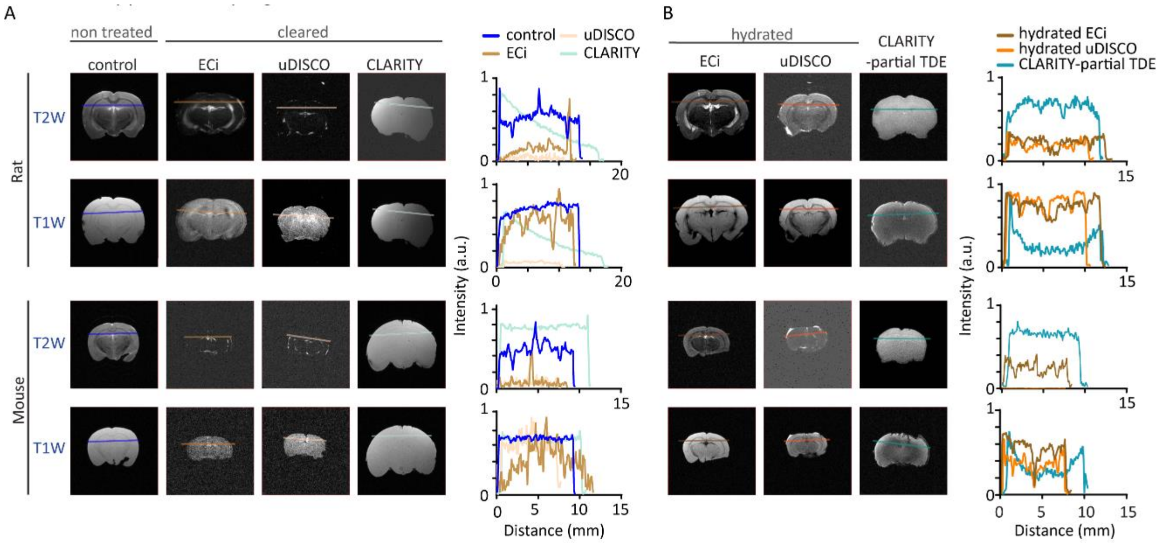

Supplementary Figure 1. Intensity profiles in coronal MRI images. A. Normalized intensity profiles from coronal T1W and T2W images of mouse and rat brains, before and after clearing by ECi, uDISCO and CLARITY, and (**B.**) following hydration of ECi and uDISCO or partial TDE treatment of CLARITY. Same images presented in Figures 1, 2 and S6.

OZ et al. Supplementary Figure 2

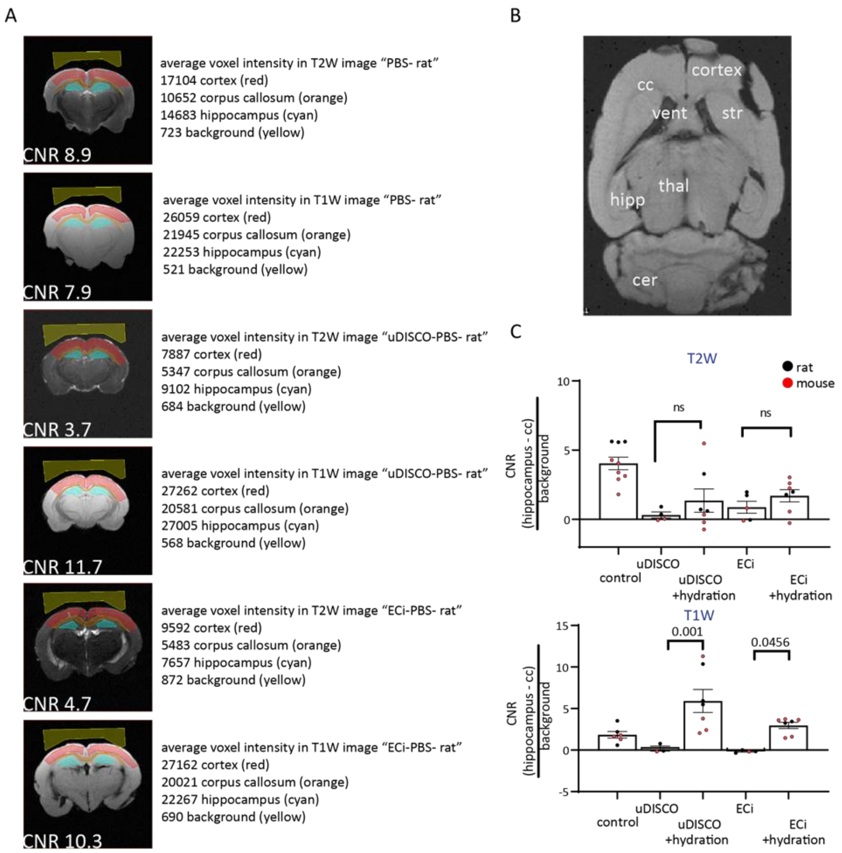

Supplementary Figure 2. Calculating CNR values representing extent of MRI-contrast. A. Representative examples of manually selected regions of interest (ROIs) and mean voxel intensities for CNR calculations. CNR is the difference between intensities of voxels in the cortex and corpus callosum, divided by background intensity. **B.** Brain regions easily seen in horizontal T1W images of hydrated ECi rat brains. Cortex, cc-corpus callosum, vent-ventricle, str-striatum, thal-thalamus, hipp-hippocampus, cer-cerebelum. **C.** CNR values calculated from the difference between signal intensities of the hippocampus (consisting of mostly grey matter) and the corpus callosum (cc, white matter), divided by the background signal. Values were taken from coronal T1W and T2W images from mouse (red) and rat (black) brains. Each point represents one brain from one animal. Data are presented as mean ±SEM. One-way ANOVA, following Sidak *post-hoc* test, was used to determine statistical significance between treatments. P-values are indicated; ***, p <0.001, n.s., non-significant.

OZ et al. Supplementary Figure 3

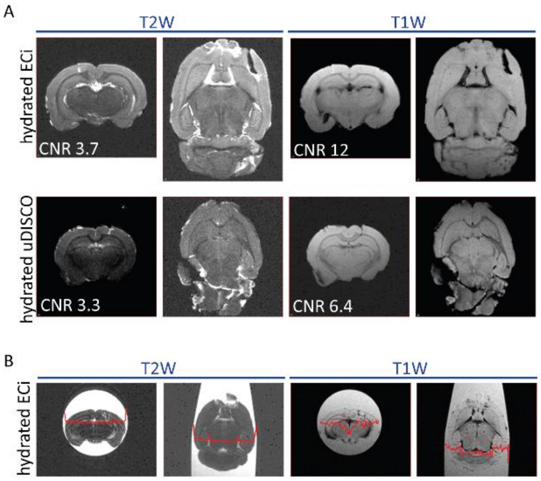

Supplementary Figure 3. The effect of hydration time and background signal on MRI-contrast. A. T2W and T1W images and CNR values from rat brains that were cleared by ECi (top) or uDISCO (bottom) following 30 days of hydration (immersion in PBS) when imaged in Fomblin Y (note black background). These are the same brain samples as shown in **Fig. 2B**. CNR was calculated as shown in **Fig. 2C**. **B.** T2W and T1W images and CNR values from hydrated ECi mouse brains when imaged in PBS. PBS elicits high background signal (see conical tube outline). Red intensity profiles show the relative intensity along the line.

OZ et al. Supplementary Figure 4

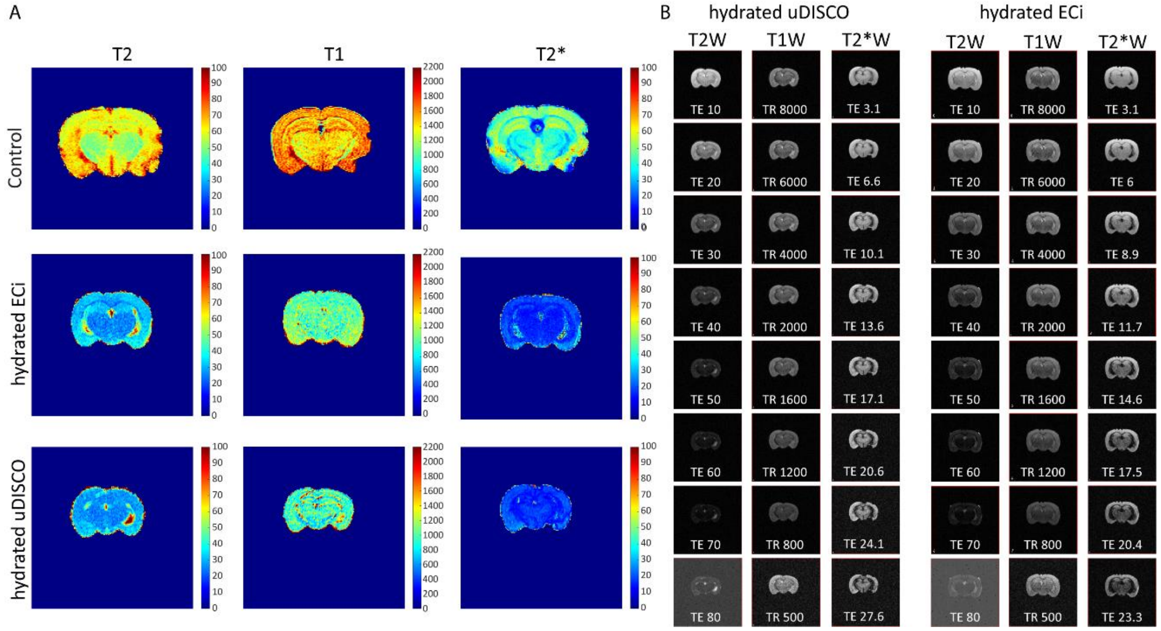

Supplementary Figure 4. Quantitative MRI. A. T1, T2 and T2*- pixel-by-pixel maps are shown for control, and hydrated ECi and uDISCO mouse brains (see **Table S1**). **B.** Individual T1, T2 and T2* -weighted images taken at variable acquisition parameters (echo time, TE; repetition time, TR) from which maps were calculated in (**A**).

OZ et al. Supplementary Figure 5

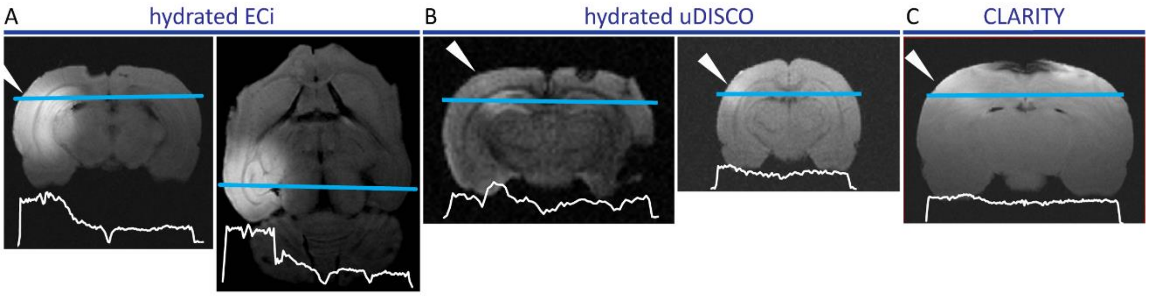

Supplementary Figure 5. GBCA injection enhances MRI-contrast in hydrated-ECi and -uDISCO samples. Cleared brains were injected with 1 µl of GBCA (0.5 mM) into left hemispheres or saline (right hemisphere), and imaged by T1W sequences (2D or 3D, see **methods**). **A.** Hydrated ECi rat brain (3D), **B.** Hydrated uDISCO mouse brain (2D, left), and rat brain (3D, right), **C.** CLARITY-rat brain (2D). Intensity profiles (white trace) show the relative intensities along a manually selected line spanning the imaged brain (blue line).

OZ et al. Supplementary Figure 6

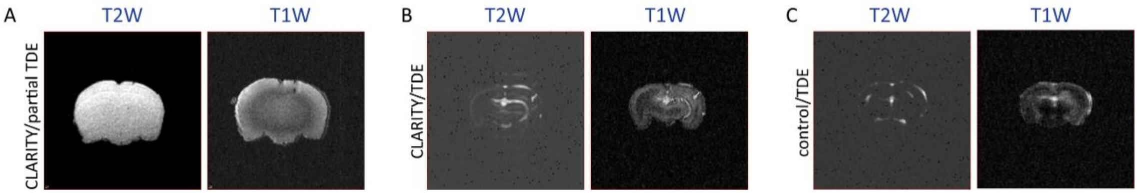

Supplementary Figure 6. Recovery of MRI-contrast in CLARITY-cleared brains. A. Coronal T1W and T2W images of mouse CLARITY-cleared brain after short incubation in TDE (partial TDE), compared to prolonged TDE incubation of CLARITY-cleared brains (**B**) or non-cleared, PFA-fixed brains (control) (**C**).

OZ et al. Supplementary Figure 7

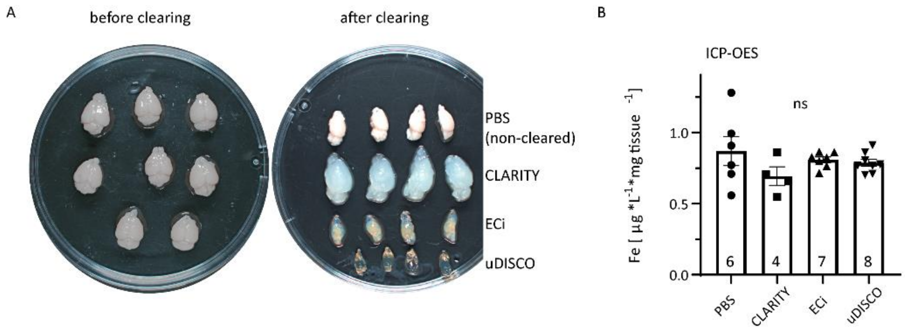

Supplementary Figure 7. Clearing by uDISCO, ECi and CLARITY does not change iron content. A. Images of PFA-fixed brains from one-month old mice (left). Each brain was divided in the midline, and each hemisphere was cleared as indicated (right image). Note the change in size of the samples due to different treatments. **B.** Iron content was quantitatively analyzed using ICP-OES. Iron concentration was normalized to the tissue weight before clearing. Number of samples are shown in bars (N=2 independent experiments). Data are shown as mean ±SEM. Statistical significance was determined by one-way ANOVA, *post-hoc* Tukey test. n.s.-non significant.

OZ et al. Supplementary Figure 8

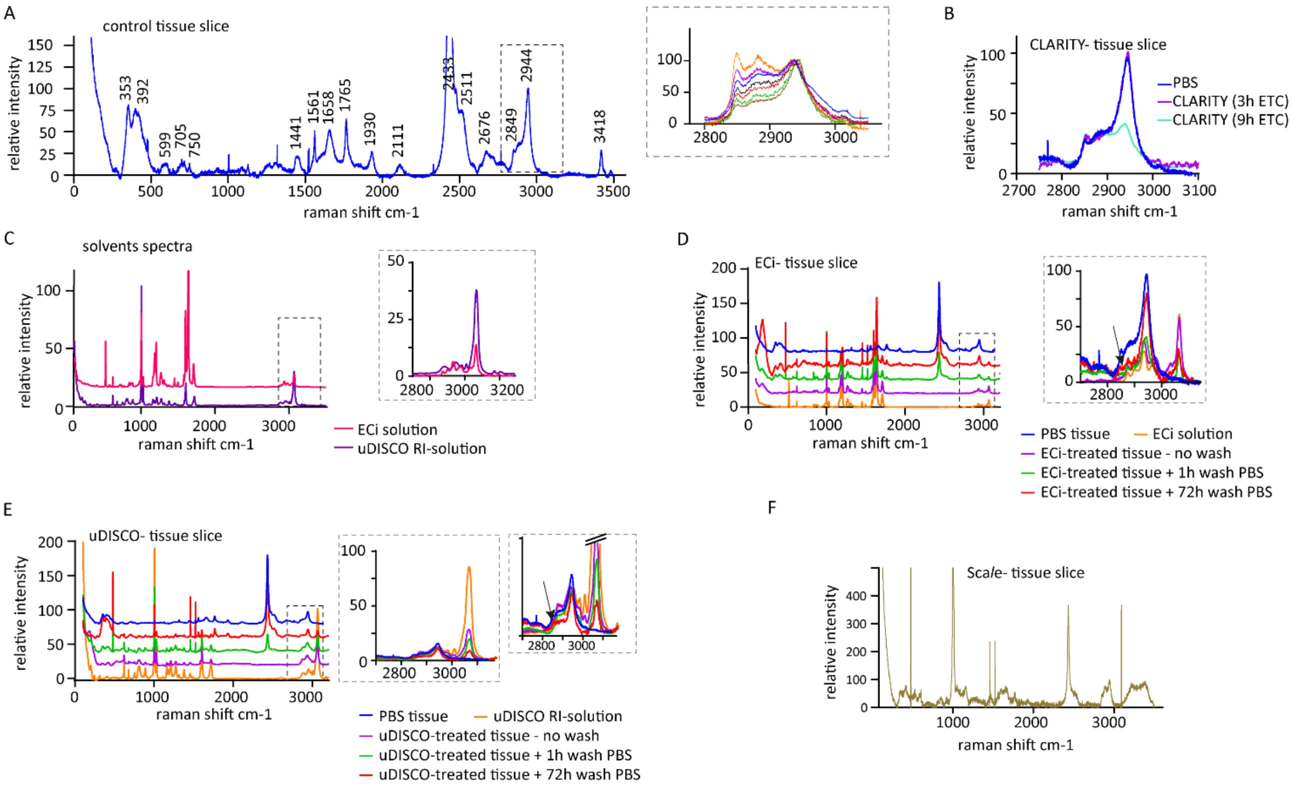

Supplementary Figure 8. Raman spectroscopy of brain slices. A. Full high resolution Raman spectrum acquired from a control (PFA-fixed) rat brain slice. Relevant peaks are numbered. Inset: Raman spectrum for a high wavenumber region, from different regions of the slice (each color represents another region in the same slice. Note the variations between the two peaks). **B.** Raman spectra from brain slices from non-treated (PBS, blue) and CLARITY cleared brains after 3 (purple) and 9 hrs (cyan) of ETC. **C.** Spectra of ECi (pink) and uDISCO RI-matching (purple) solutions and zoom-in (inset) of the high wavenumber region. **D.** Extensive washing does not remove ECi solvents. Raman spectra of brain slices from non-treated (PBS, blue trace), ECi solution (orange), and ECi-cleared samples in ECi solution (no wash, purple) and following wash by PBS for 1 (green) or 72 hrs (red). Inset: Magnification of the high wavenumber region. **E.** Raman spectra from brain slices from non-treated (PBS, blue trace), uDISCO RI-matching solution (orange), and uDISCO-cleared samples (in uDISCO RI-matching solution, purple) and following wash by PBS for 1 (green)

or 72 hrs (red). Inset: Magnification of the high wavenumber regions. **F.** Raman spectrum of a Sca*le*-cleared brain tissue.

OZ et al. Supplementary Figure 9

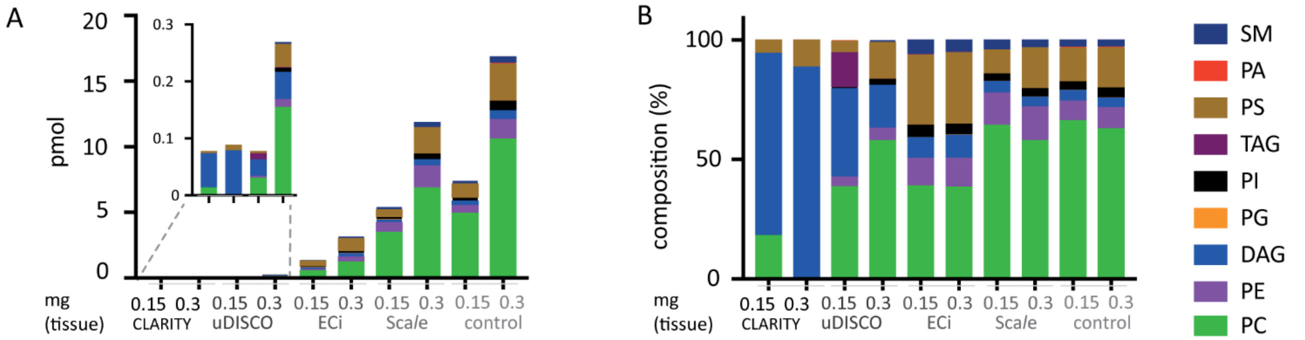

Supplementary Figure 9. Quantitative lipidomics analysis. Two samples of 150 and 300 µg were taken from each homogenate. **A.** Total lipid content (pmol) in control (non-treated), Sca*le*, CLARITY, uDISCO and ECi -cleared brains homogenates. **B.** Relative composition of lipid subtypes (% from total). PA - phosphatidate, PC - phosphatidylcholine, PE - phosphatidylethanolamine, PG - phosphatidylglycerol, PI - phosphatidylinositol, PS - phosphatidylserine, DAG - diacylglycerol, TAG - triacylglycerol, SM - sphingomyelin.

OZ et al. Supplementary Figure 10

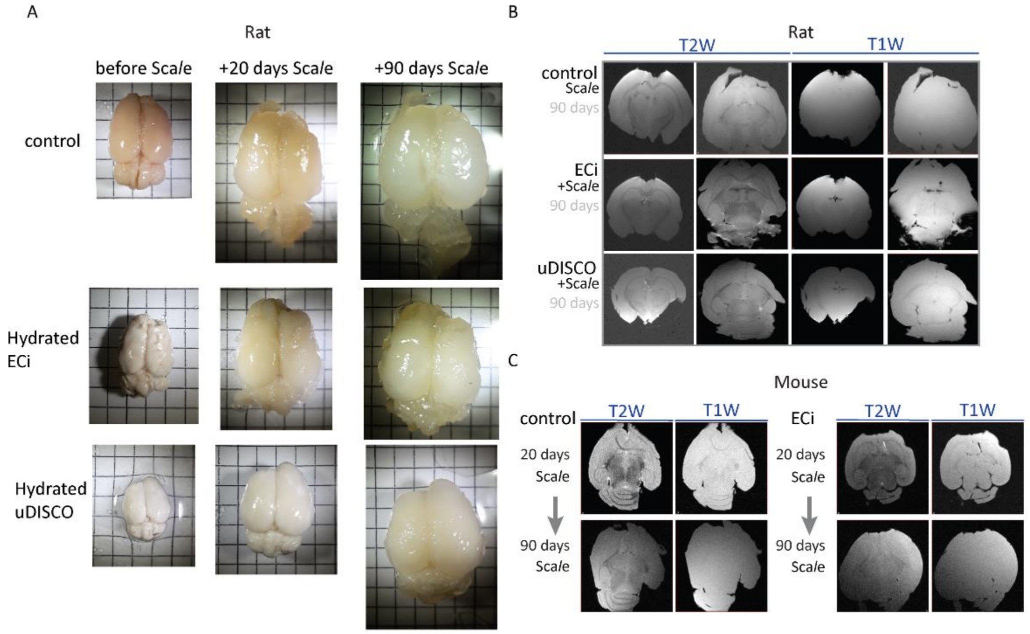

Supplementary Figure 10. Changes in appearance and loss of MRI-contrast following Sca*le*-clearing. A. Representative images of PFA-fixed (control, top row), ECi- (middle) and uDISCO-cleared rat brains

(bottom) undergoing progressive changes in appearance, following 20 and 90 days treatment by the Sca*l*e reagents. Samples are imaged on a 0.5 cm grid paper, without immersion in a RI-matching solution. **B.** Coronal T1W and T2W images of control, ECi and uDISCO-cleared rat brains after these have been treated by the Sca*l*e reagents at indicated times (grey). **C.** Horizontal T1W and T2W images of control and ECi-cleared mouse brains following 20 and 90 days treatment in Sca*l*e reagents.

OZ et al. Supplementary Figure 11

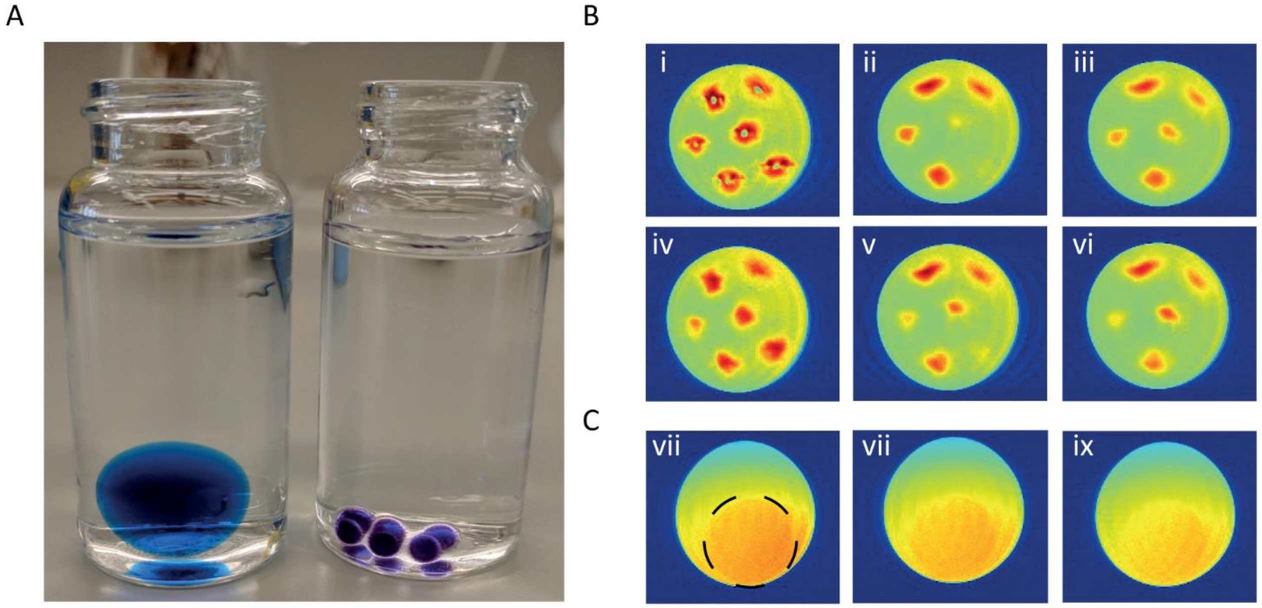

Supplementary figure 11. MRI of water-absorbing polymer beads. A. Water absorbing gel beads were placed in a water filled tube (right, purple beads) resulting in their immediate water absorbance and swell (left, blue). **B.** Longitudinal T2W MR images show expansion of six beads and concomitant reduction in MRI-signal, until the complete loss of MRI-contrast (**C**, dashed circle). For clarity, in C we imaged a single bead at the end of the process, as the excessively swollen beads occupied a larger volume than the tube's and could not be imaged together.

Table S1. Longitudinal (T1) and transverse (T2/T2*) relaxation times values are mean ± SD of the entire slice (A) or specific regions (B).

A.

	**T2 (ms)**	**T1 (ms)**	**T2* (ms)**
**PBS**	61 ± 3	1565 ± 384	45 ± 17
**Hydrated ECi**	32 ± 12	1047 ± 189	20 ± 21
**Hydrated uDISCO**	31 ± 13	1014 ± 310	18 ± 5
**PBS/TDE/PBS**	35 ± 13	832 ± 264	24 ± 7
**CLARITY/TDE/PBS**	83 ± 11	1700 ± 492	85 ± 24
**CLARITY/TDE/GBCA**	23 ± 4	131 ± 28	11 ± 4

B.

	**T2 (ms)**	**T1 (ms)**	**T2* (ms)**
		**PBS**	
**cortex**	59 ± 4	1573 ± 127	50 ± 4
**cc**	63 ± 4	1342 ± 185	38 ± 8
**hippocampus**	59 ± 4	1514 ± 176	52 ± 5
		**Hydrated ECi**	
**cortex**	31 ± 3	999 ± 130	19 ± 2
**cc**	26 ± 4	955 ± 145	15 ± 2
**hippocampus**	32 ± 4	1075 ± 128	16 ± 3
		**Hydrated uDISCO**	
**cortex**	29 ± 5	1003 ± 221	19 ± 3
**cc**	26 ± 4	814 ± 187	14 ± 2
**hippocampus**	27 ± 3	999 ± 206	16 ± 4
